# Long-range chemical signalling in vivo is regulated by mechanical signals

**DOI:** 10.1038/s41563-025-02463-9

**Published:** 2026-01-19

**Authors:** Eva K. Pillai, Sudipta Mukherjee, Niklas Gampl, Ross J. McGinn, Katrin A. Mooslehner, Julia M. Becker, Alexander K. Winkel, Amelia J. Thompson, Kristian Franze

**Affiliations:** 1 Department of Physiology, Development and Neuroscience, University of Cambridge, Cambridge, UK; 2 Cell Biology and Biophysics Unit, European Molecular Biology Laboratory, Heidelberg, Germany; 3 Developmental Biology Unit, European Molecular Biology Laboratory, Heidelberg, Germany; 4 Medical Institute of Biophysics, Friedrich-Alexander-Universität Erlangen-Nürnberg, Erlangen, Germany; 5 Max-Planck-Zentrum für Physik und Medizin, Erlangen, Germany; 6 Wellcome–MRC Cambridge Stem Cell Institute, Jeffrey Cheah Biomedical Centre, Cambridge Biomedical Campus, Cambridge, UK

**Keywords:** Developmental biology, Biophysics

## Abstract

Biological processes are regulated by chemical and mechanical signals, yet how these signalling modalities interact remains poorly understood. Here we identify a crosstalk between tissue stiffness and long-range chemical signalling in the developing *Xenopus laevis* brain. Targeted knockdown of the mechanosensitive ion channel Piezo1 in retinal ganglion cells or in the brain tissue surrounding retinal ganglion cells causes pathfinding errors in vivo. In the brain parenchyma, Piezo1 downregulation decreases the expression of the diffusive long-range chemical guidance cues Semaphorin3A (Sema3A) and Slit1, which instruct turning responses in distant cells. Furthermore, Piezo1 knockdown results in tissue softening due to reduced expression of the adhesion proteins NCAM1 and N-cadherin. Targeted depletion of NCAM1 and N-cadherin similarly reduces tissue stiffness and Sema3A expression. Conversely, increasing environmental stiffness ex vivo enhances tissue-level force generation and Slit1 and Sema3A expression. Finally, in vivo stiffening of soft brain regions induces ectopic Sema3A production via a Piezo1-dependent mechanism. Overall, these findings demonstrate that tissue mechanics locally modulates the availability of diffusive, long-range chemical signals, thus influencing cell function at sites distant from the mechanical cue.

## Abstract (web summary)

Tissue stiffness mediated by Piezo1 is shown to regulate the expression of diffusive guidance cues in the developing *Xenopus laevis* brain, revealing a crosstalk between mechanical signals and long-range chemical signalling.

## Main

Numerous biological processes are regulated by long-range concentration gradients of diffusible chemical signals, which act in concert to spatiotemporally control the function of cells within tissues^1^. Morphogen gradients, for example, set positional information during development^2–6^, while growth factor and hormone gradients are essential for organ development and maintenance^7^. Signalling molecules have multitudinous functions; for instance, the Semaphorin family of proteins regulate cell morphology and motility in the nervous, immune, respiratory, cardiovascular and musculoskeletal systems during development, homeostasis and disease^8^. Similarly, Slit proteins affect brain, kidney, heart and mammary gland development, and play roles in various disease states, by regulating cell proliferation, migration, vascularization and more^9^.

Recent work demonstrated that many of these cellular functions are also regulated by tissue mechanics. For instance, mechanical anisotropies in tissues affect growth orientation, while factors such as mechanical stresses, environmental stiffness, and cell or tissue geometry profoundly affect cell proliferation, migration and fate specification^10–12^. In the nervous system, for example, mechanical forces regulate development and various physiological processes, while alterations in tissue mechanics occur in neurodegenerative diseases and other pathophysiological conditions^13^. Thus, besides the well-established role of chemical signals, mechanical cues are equally critical regulators of biological processes.

**Fig. 1:**
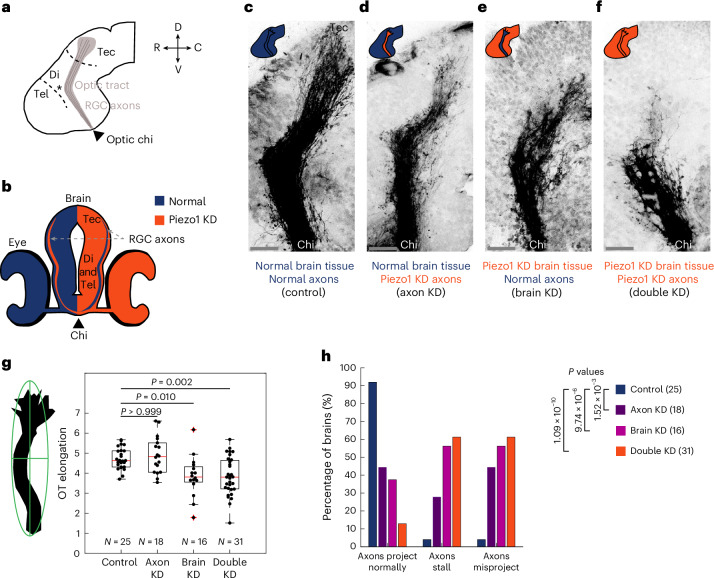
RGC axon pathfinding in vivo requires cell-intrinsic and cell-extrinsic Piezo1 signalling. **a**, Schematic of the lateral view of a stage 40 *Xenopus* brain with orientation guides for rostral–caudal (R, C) and dorsal–ventral (D, V) axes. RGC axons grow from the optic chiasm towards the optic tectum along a stereotypic path (the optic tract), turning caudally at the mid-diencephalon (marked by an asterisk). **b**, Schematic cross-section of a *Xenopus* brain and retinae when Piezo1 is downregulated unilaterally in the nervous system. RGC axons cross the midline at the optic chiasm and grow across the contralateral brain surface. Normal Piezo1 levels are indicated in blue while Piezo1 depletion is shown in red. Piezo1-depleted axons grow across brain tissue with normal Piezo1 levels and vice versa. **c**–**f**, Images of RGC axon growth in vivo in control (**c**), Piezo1-depleted axons (**d**), Piezo1-depleted surrounding brain tissue (parenchyma) (**e**), and both axons and brain tissue depleted of Piezo1 (**f**). Scale bars, 50 μm. **g**, Optic tract (OT) elongation. Schematic representation of the fitted ellipse used to determine optic tract elongation, expressed as the ratio of long to short axes. Quantification for the indicated conditions (Kruskal–Wallis test, *P* < 0.0001, followed by Dunn’s post hoc test; adjusted *P*-values indicated). Each point represents a brain. Boxes show median, first and third quartiles; whiskers show the spread of data; ‘+’ indicate outliers. *N* denotes the number of animals. **h**, Scoring of brains displaying aberrant phenotypes. Quantification of embryos showing normal, stalling or misprojection defects after Piezo1 knockdown in axons, brain tissue or both (two-tailed chi-squared test, *P* = 1.014 × 10^−8^, followed by Fisher’s exact post hoc tests; number of animals indicated in parentheses) (Extended Data Fig. 2). Data are pooled from a minimum of three independent experiments. chi, chiasm; di, diencephalon; KD, knockdown; tec, tectum; tel, telencephalon.

Cells will always be simultaneously exposed to both chemical and mechanical cues. One example is the extension of neuronal axons along precise paths during nervous system development, termed axon pathfinding. In the *Xenopus laevis* optic pathway, retinal ganglion cell (RGC) axons exhibit a stereotypical trajectory, exiting the eye via the optic nerve, crossing the midline at the optic chiasm, traversing the contralateral brain surface, and making a characteristic caudal turn at the mid-diencephalon before reaching their target, the optic tectum^14,15^ (Fig. 1a). Several membrane-bound and diffusible signalling molecules pattern the surrounding brain tissue, directing *Xenopus* RGC axons along this specific path^16^. For instance, the caudal turn of RGC axons in the mid-diencephalon is regulated by diffusive gradients of the repulsive long-range guidance cues, Slit1^17^ and Semaphorin3A (Sema3A)^18^. At the same time, RGC axons are mechanosensitive and encounter an instructive stiffness gradient in the brain, which also contributes to regulating RGC axon pathfinding in that region^19,20^. However, little is currently known about how cells integrate chemical and mechanical signalling in vivo. While recent studies exquisitely showed how environmental mechanics may regulate cell-intrinsic gene expression patterns and chemical signalling pathways^21–24^, how mechanical cues influence cell-extrinsic signalling—and, consequently, the function of distant cells not directly exposed to the mechanical signal—remains elusive.

## Piezo1 regulates axon pathfinding cell-intrinsically and cell-extrinsically

A key player involved in sensing and responding to brain tissue stiffness^19,25^ is the mechanosensitive non-selective cation channel Piezo1^26,27^. Piezo1 is found in a broad range of animal tissues, including the bladder, kidneys, lung and skin^26^, and in the developing *Xenopus* nervous system^19,28^. Downregulating Piezo1 expression in the nervous system in vivo leads to aberrant axon growth and severe pathfinding defects during development^19^. To disentangle the effect of Piezo1 activity in RGC axons from that of the surrounding neuroepithelial cells on axon pathfinding, we selectively downregulated Piezo1 in the axons while preserving its expression in the surrounding brain tissue.

**Extended Data Fig. 1:**
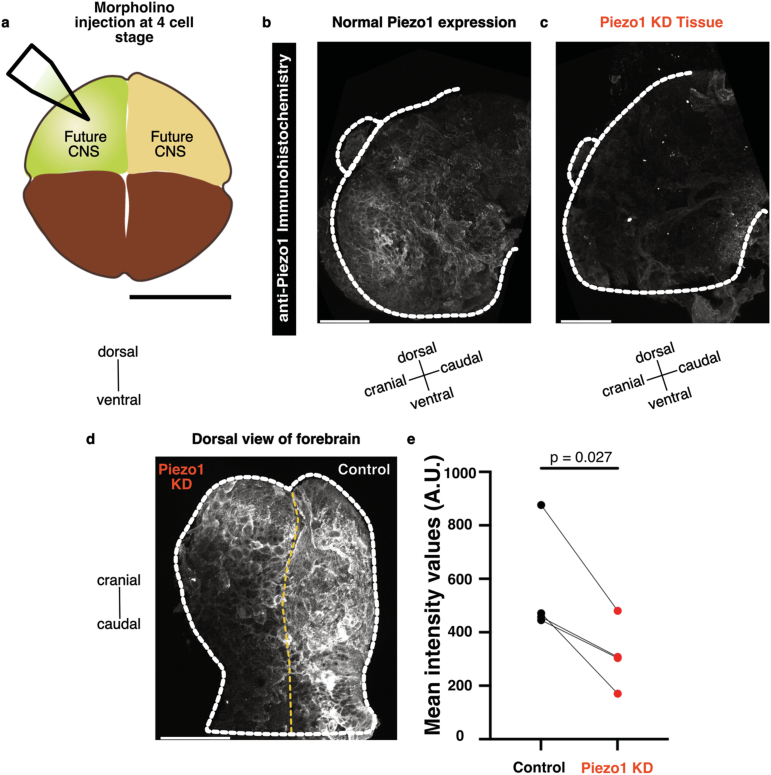
Downregulation of Piezo1 in half of the developing neuroepithelium. **a**, Schematic. At the 4-cell stage, translation-blocking morpholinos were injected into one of the two dorsal (usually less pigmented) blastomeres, which contribute to the formation of the nervous system. Scale bar: 600 µm. **b-d**, Immunofluorescence images of *Xenopus laevis* brains at stage 40. The dashed white outlines depict the boundaries of the brain tissue. Scale bars: 100 µm. **b-c**, Piezo1 expression patterns in neuroepithelium (lateral view) arising from the **b**, uninjected blastomere and **c**, the blastomere injected with translation-blocking Piezo1 morpholino, resulting in Piezo1 knockdown (KD). **d**, Dorsal view of Piezo1 expression in the developing neuroepithelium showing both the injected (Piezo1 KD) and the uninjected (Control) hemispheres. The dashed yellow line represents the midline. **e**, Piezo1 morpholino injection into one dorsal blastomere led to a significant decrease in Piezo1 expression in one half of the developing neuroepithelium. The graph represents the paired (Control vs Piezo1 KD) mean intensity values of the developing telencephalon. Each point represents an embryo (*N* = 4). Data was assessed with a two tailed ratio paired t-test; *p*-value indicated in the figure. KD: knockdown.

Classical fate-mapping experiments have shown that the two dorsal blastomeres of four-cell-stage embryos contribute to the formation of the nervous system, with each blastomere contributing to one half of it^29,30^. Injecting translation-blocking morpholinos into one of the two dorsal blastomeres at the four-cell stage (Extended Data Fig. 1a) therefore resulted in embryos with Piezo1 depletion in one half of the nervous system. In pre-metamorphic *Xenopus* embryos, all RGC axons cross the midline at the optic chiasm to the contralateral surface, without forming ipsilateral projections^31^ (which are typically only formed from stage 52^32^). Thus, this approach yielded embryos with Piezo1-depleted RGC axons growing across healthy brain tissue, while in the contralateral brain hemisphere, normal RGC axons grew across Piezo1-depleted brain tissue (Fig. 1b and Extended Data Fig. 1). While it was challenging to confirm Piezo1 depletion specifically in RGC axons in vivo, as Piezo1 is expressed not only in the axons^19^ but also ubiquitously throughout the surrounding brain parenchyma, local Piezo1 depletion was clearly visible in the parenchyma of the tissue arising from the injected blastomere (Extended Data Fig. 1).

**Extended Data Fig. 2:**
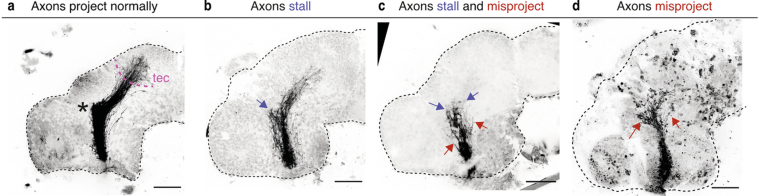
RGC axon growth phenotypes and guidance defects in the developing *Xenopus* brain. **a**, Normal RGC axonal projections in the brain of a stage 40 *Xenopus laevis* embryo. RGC axons form a bundle, turn in the mid-diencephalon (marked with ‘*’), and arrive at the optic tectum (tec). **b**, Representative image of a brain showing stalling of some RGC axons (purple arrow) before others turn caudally in the mid-diencephalon. **c**, Representative image of a brain showing RGC axon stalling (purple arrows) and splaying (that is, unbundling / defasciculation), leading to misprojections (red arrows). **d**, Representative image of a brain showing RGC axons unbundling and misprojecting (red arrows). Scale bars: 100μm. RGC: retinal ganglion cell.

RGC axons were labelled with DiI, and their trajectories analysed at developmental stage 40^33^, when axons had reached their end target, the optic tectum. Consistent with our previous findings^19^, simultaneous downregulation of Piezo1 in both axons and the surrounding brain tissue led to severe axon pathfinding defects (Fig. 1c,f), compared with age-matched sibling embryos, where both dorsal blastomeres were injected with a control morpholino. The elongation of the RGC axon bundle growing along the brain surface, or optic tract, was significantly lower than in controls (*P* = 0.002, Kruskal–Wallis with Dunn’s post hoc test) (Fig. 1g). Furthermore, only 13% of brains had normal axonal projections, while 61% of brains exhibited stalling and/or misprojection defects (that is, deviations from the normal trajectory, such as bypassing the caudal turn in the mid-diencephalon or misrouting dorsoanteriorly into the telencephalon) (Fig. 1h). Representative images illustrating normal RGC axonal projections and various axon guidance defects are shown in Extended Data Fig. 2.

When Piezo1 was selectively depleted in RGC axons (Fig. 1d), optic tract elongation was similar as in controls (*P* > 0.999, Kruskal–Wallis with Dunn’s post hoc test, Fig. 1g). Nonetheless, only 44% of these brains exhibited normal axonal projections (Fig. 1h). Piezo1 downregulation in the axons resulted in 28% of brains with stalling defects, while 44% of the brains showed misprojecting or both stalling and misprojecting defects (Fig. 1h). These results confirmed that RGC axons require Piezo1 to respond to their mechanical environment, and that this cell-intrinsic mechanosensing is necessary for accurate pathfinding. However, the overall phenotype was milder than what we had observed in brains with downregulated Piezo1 expression in both RGC axons and the surrounding neuroepithelium^19^ (Fig. 1f–h), suggesting that Piezo1 expression in neuroepithelial cells was also required for proper axon pathfinding.

Indeed, when Piezo1 downregulation was limited to the surrounding brain tissue, axon pathfinding of healthy RGCs containing Piezo1 was also perturbed. In fact, the observed guidance defects were more profound than in brains with only axonal Piezo1 knockdown (Fig. 1e). Optic tract elongation was significantly decreased when compared to controls (*P* = 0.010, Kruskal–Wallis with Dunn’s post hoc test) (Fig. 1g), and only 38% of brains showed normal axonal projections. In contrast, 56% of brains exhibited stalling and/or splaying (that is, unbundling) and misprojecting axons (Fig. 1h). Thus, despite normal Piezo1 levels in RGC axons, axon growth patterns were disrupted, confirming that Piezo1-mediated mechanosensing by neuroepithelial cells was also essential for accurate axon pathfinding.

## Piezo1 regulates expression of long-range guidance cues in vivo

**Fig. 2:**
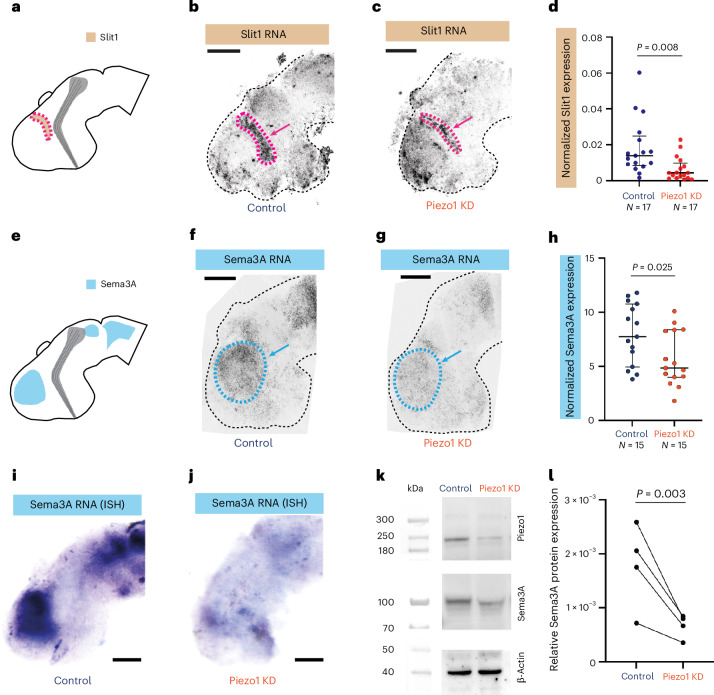
Piezo1 downregulation attenuates the expression of diffusive long-range guidance cues in vivo*.* **a**, Schematic representation of the expression pattern of the diffusive chemical guidance cue, Slit1. **b**,**c**, Representative HCR-FISH images of Slit1 expression in control (**b**) and Piezo1 knockdown (**c**) brains. **d**, Quantification of Slit1 mRNA expression (two-tailed unpaired *t*-test with Welch’s correction; *P* value indicated). **e**, Schematic of the Sema3A expression pattern. **f**,**g**, Representative HCR-FISH images of Sema3A expression in control (**f**) and Piezo1 knockdown (**g**) brains. **h**, Quantification of normalized Sema3A expression (two-tailed unpaired t-test with Welch’s correction; *P* value indicated). **i**,**j**, In situ hybridization of Sema3A mRNA expression in control (**i**) and Piezo1 knockdown (**j**) brains. **k**, Western blot of Piezo1, Sema3A and β-actin protein expression in control and Piezo1-depleted brains. **l**, Western blot quantification (*N* = 4, normalized to total protein, each point indicates the mean value of a biological replicate; two-tailed ratio paired *t*-test; *P* value indicated). Each point in **d**,**h** represents an embryo; bars indicate lower quartile, median and upper quartiles. *N*, number of animals. Scale bars, 100 μm. ISH, in situ hybridization.

We next investigated how perturbations of neuroepithelial mechanosensing may lead to disruptions in growth patterns of healthy axons. At least two RGC-extrinsic factors are crucial for the turning of the optic tract in the mid-diencephalon (Fig. 1a): local tissue stiffness^19^ and long-range chemical signalling by the repulsive guidance cues Slit1^17^ and Sema3A^18^, which are both located rostrally to the optic tract’s caudal turn (Fig. 2a,e). Chemical perturbations of brain tissue stiffness in vivo mostly lead to a splaying of RGC axons in the diencephalon^19^, while depletion of Slit1 and Sema3A signalling predominantly leads to a stalled axon phenotype^17,34^. Since we found both axon guidance defects after downregulating Piezo1 expression in neuroepithelial cells (Fig. 1e,h), we first tested the effect of Piezo1 downregulation on the expression of these chemical guidance cues using hybridization chain reaction-fluorescence in situ hybridization (HCR-FISH).

**Extended Data Fig. 3:**
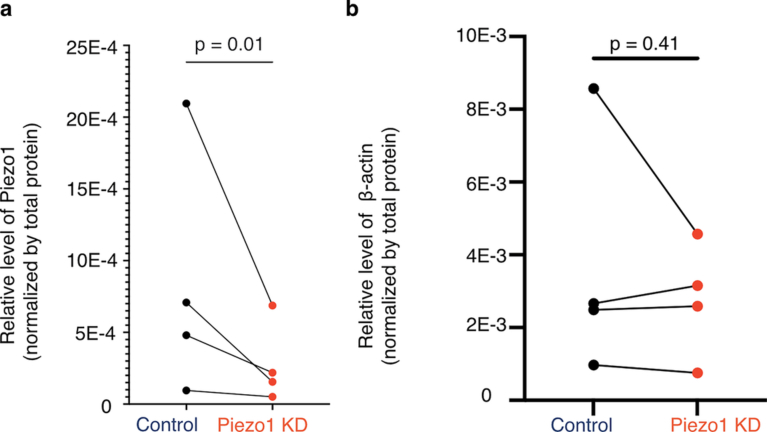
Quantification of Western blots. Downregulating Piezo1 led to a significant decrease in the expression of Piezo1 protein **a**, but not of β-actin **b**. Representative Western blot images are shown in Fig. 2k. Data were normalised by the total protein concentration. Each point represents the mean of a biological replicate (N = 4; two-tailed ratio paired t-test; p-values indicated). KD: knockdown.

In stage 40 control injected sibling embryos, our HCR-FISH results recapitulate published in situ hybridization data^17,18,35^: Slit1 forms a distinct band between the telencephalon and diencephalon, while Sema3A is highly expressed rostroventrally of Slit1 in the telencephalon (Fig. 2b,f). Piezo1 downregulation resulted in a significant reduction in Slit1 mRNA expression (*P* = 0.008, unpaired *t*-test with Welch’s correction) (Fig. 2c,d), and Sema3A mRNA expression was significantly decreased compared to controls (*P* = 0.025, unpaired *t*-test with Welch’s correction) (Fig. 2g,h). Standard in situ hybridizations confirmed the dramatic decrease in Sema3A mRNA expression in Piezo1-depleted brain tissue (Fig. 2i,j). Additionally, Western blot analysis confirmed that Piezo1 downregulation resulted in significant decreases in Sema3A (*P* = 0.003, ratio paired *t*-test) (Fig. 2k,l) and Piezo1 protein levels, but not in the housekeeping protein β-actin (Fig. 2k and Extended Data Fig. 3); Slit1 protein expression could not be assessed due to a lack of suitable antibodies in *Xenopus*. Hence, our data indicated that the downregulation of Piezo1—a mechanosensor—in neuroepithelial cells attenuated the expression of long-range chemical cues, implying a role for mechanical signalling in modulating chemical signalling during development.

## Piezo1, but not Sema3A, depletion leads to brain tissue softening

**Fig. 3:**
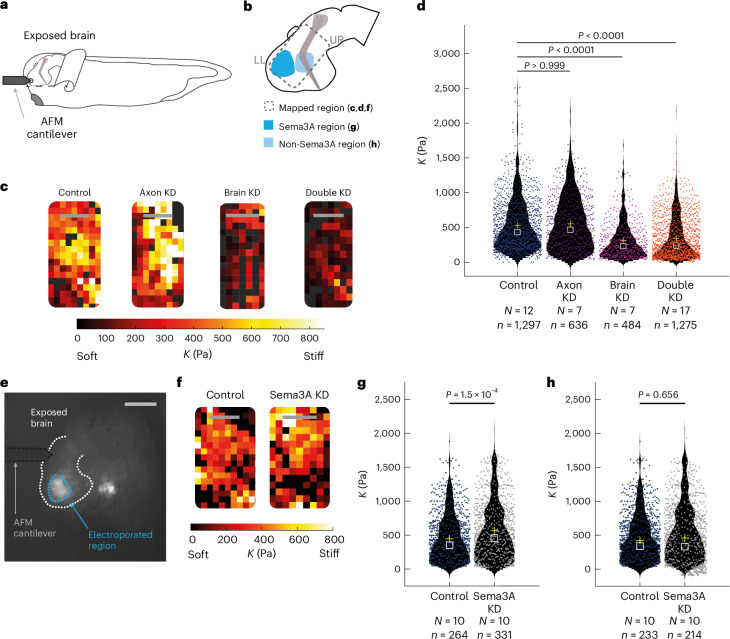
Piezo1, but not Sema3A, knockdown leads to brain tissue softening. **a**, Schematic of the experimental set-up for in vivo brain-stiffness mapping. **b**, *Xenopus* brain schematics. Dashed rectangle indicates the mapped region; lower left (LL) and upper right (UR) corners of stiffness maps (**c**,**f**) are indicated, colours indicate areas selected for regional analysis (**g**,**h**). **c**, AFM-based stiffness maps (colour maps) encoding the apparent elastic modulus, *K*, a measure of tissue stiffness, assessed at an indentation force *F* = 10 nN for control or Piezo1 downregulation exclusively in axons, in the surrounding brain tissue, or both. **d**, Quantification of AFM measurements of stiffness in different conditions (Kruskal–Wallis test, *P* < 0.0001; Dunn’s post hoc tests, adjusted *P* values indicated). **e**, Exposed brain of stage 40 *Xenopus* embryo electroporated with fluorescein-tagged morpholinos to visualize electroporated regions. Dashed lines: brain outline (white), electroporated region (blue) and AFM cantilever (black). Scale bar, 250 µm. **f**–**h**, Downregulating Sema3A and assessing tissue stiffness: AFM-based stiffness maps for control or Sema3A morpholino-electroporated brains (**f**); quantification of AFM stiffness measurements in Sema3A-producing regions (**g**) and adjacent non-Sema3A-producing regions (**h**) (Wilcoxon rank-sum test, *P* values indicated). Violin plots in **d**,**g**,**h** display the data distribution, overlayed with individual measurements shown as scattered points; means and medians are indicated as yellow crosses and white squares, respectively. Scale bars, 100 μm. *N*, number of animals; *n*, number of measurements.

The splaying of axons seen in Piezo1-downregulated brain parenchyma (Fig. 1e,f) recapitulated the phenotype of RGC axons growing through brain tissue that was chemically softened in vivo^19^. To test if, in addition to the chemical landscape, tissue mechanics was also altered in Piezo1 knockdown brains, we used atomic force microscopy (AFM)-based stiffness mapping^19,20^ to measure the stiffness of live developing *Xenopus* brains at stage 40. Tissue stiffness was quantified by the reduced apparent elastic modulus, *K*, whereby a larger *K* value indicates stiffer tissue. Measurements were performed in vivo in a rectangular grid on the exposed brain surface (Fig. 3a,b) of stage-matched sibling embryos for control injected embryos and embryos in which Piezo1 was downregulated either exclusively in the axons or in the surrounding brain tissue, or in both. We have previously shown that RGC axons do not contribute significantly to brain tissue stiffness^19^; thus, variations in axon growth are unlikely to affect the measured stiffness here.

The overall mechanical landscape of brains with Piezo1 downregulation in solely the RGC axons was similar to that of control embryos (median *K*_ctrl_ = 432 Pa, *K*_a__xon__K/D_ = 467 Pa; *P* > 0.999, Kruskal–Wallis with Dunn’s post hoc test) (Fig. 3c,d), suggesting that the observed axon pathfinding defects shown in Fig. 1d were not a consequence of alterations in tissue stiffness but rather originated from the axons’ reduced ability to sense their mechanical environment. Conversely, downregulating Piezo1 either exclusively in the surrounding brain tissue or simultaneously in the axons and surrounding brain tissue resulted in a close to two-fold decrease in brain stiffness (median *K*_brain K/D_ = 231 Pa, *K*_double K/D_ = 240 Pa; *P* < 0.0001, Kruskal–Wallis with Dunn’s post hoc test) (Fig. 3c,d). Hence, the knockdown of Piezo1 in the developing neuroepithelium led to considerable changes in both the chemical and mechanical landscape encountered by growing RGC axons.

**Extended Data Fig. 4:**
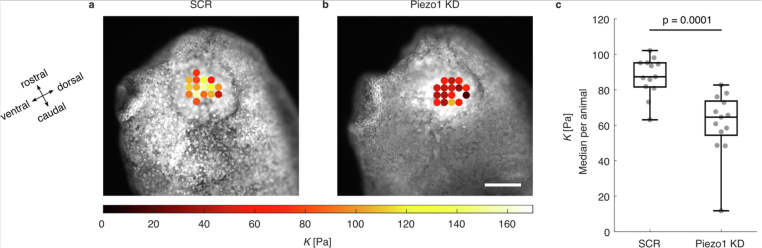
Piezo1 knockdown leads to softening of skin tissue. **a, b**, Heatmaps of AFM measurements for stage 31 **a**, SCR (control) and **b**, Piezo1 knockdown (KD) embryo skin tissue, overlaid on a fluorescence image of the GFP-containing injected constructs. Heatmaps are scaled to an apparent elastic modulus of 170 Pa; scale bar is 200 μm. **c**, Median skin tissue stiffness per animal in both conditions. Each dot corresponds to one animal; boxes span the first and third quartiles with a line at the median. The whiskers span the range of the data. Both groups were compared with a nested t-test, where AFM measurements were nested under animals, and animals under condition. N = 13 animals per condition; n = 3 - 23 stiffness measurements per animal.

To test whether the observed tissue softening following knockdown of Piezo1 was specific to the central nervous system or a more general phenomenon, we also measured the stiffness of skin in *Xenopus* embryos at stages 28–31 using AFM. Here too, Piezo1 knockdown led to a significant softening of the tissue (Extended Data Fig. 4), indicating that Piezo1 may not only sense but also regulate tissue stiffness in other organ systems.

We then wanted to know whether there was a causal relationship between the decrease in guidance cue expression (Fig. 2) and the decrease in tissue stiffness (Fig. 3c,d) in Piezo1 knockdown brains. In particular, we asked if tissue softening was a consequence of the decrease in Sema3A and Slit1, or if, conversely, the decrease in guidance cue expression was a consequence of the change in tissue stiffness. Given the spatially restricted expression of Slit1 mRNA and the uncertainty if protein levels had changed after Piezo1 knockdown, we here focused on the effect of Sema3A depletion on tissue stiffness.

To downregulate endogenous Sema3A protein in the brain without altering the early developmental expression of Piezo1 levels, we electroporated the forebrain of stage 29/30 embryos with either a Sema3A or a control fluorescein-tagged morpholino^34^. The electroporated embryos were allowed to develop to stage 40, at which point tissue stiffness was measured using AFM (Fig. 3e). Downregulating Sema3A protein expression in the forebrain did not result in brain tissue softening. In fact, the stiffness of the Sema3A-depleted forebrain region increased compared to controls, with a median *K* = 455 Pa in the Sema3A knockdown brain and *K* = 350 Pa in the control (*P* < 0.0001, Wilcoxon rank-sum test) (Fig. 3f,g), while brain tissue mechanics in adjacent regions was unaltered in these embryos (median *K*_Sema3A K/D_ = 332 Pa, *K*_ctrl_ = 341 Pa; *P* = 0.67, Wilcoxon rank-sum test) (Fig. 3h). These data showed that the observed stiffening was specific to the localized Sema3A depletion, and suggested that the decrease in guidance cues in Piezo1 knockdown brains is probably not responsible for the decrease in tissue stiffness.

## Brain tissue softens due to decreased cell–cell adhesion

**Fig. 4:**
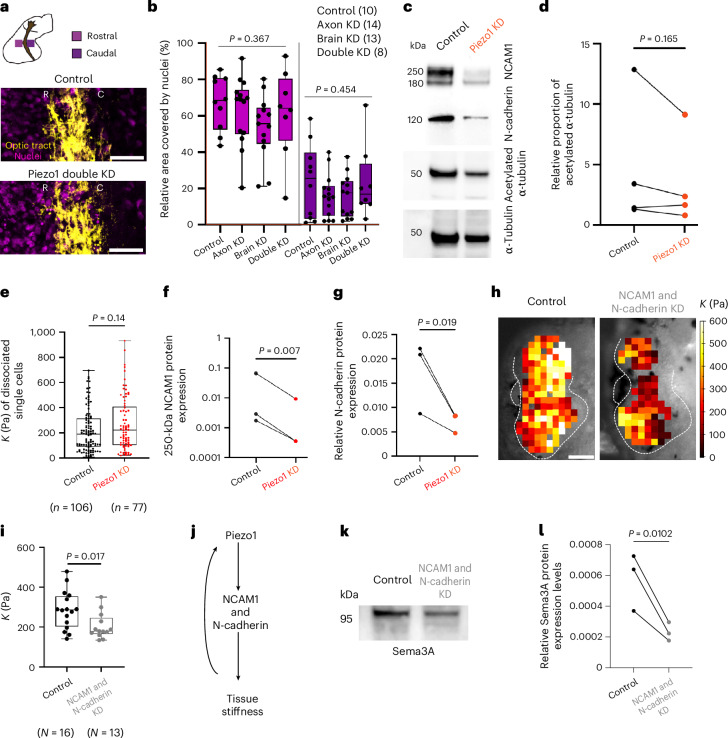
Piezo1 modulates tissue stiffness by regulating cell–cell adhesion. **a**,**b**, Local cell body densities. **a**, Representative images of nuclei (DAPI, magenta) in brain tissue rostral (R) and caudal (C) to the optic tract (DiI, yellow) in stage 40 *Xenopus* embryos. Scale bars, 50 μm. **b**, Relative nuclear area rostral and caudal to the optic tract (two independent experiments; one-way ANOVA). **c**, Western blot of NCAM1, N-cadherin, acetylated α-tubulin and total α-tubulin in control and Piezo1-depleted brains. **d**, Western blot quantification (*N* = 4) of relative proportion of acetylated α tubulin (ratio of acetylated α-tubulin/total α-tubulin). **e**, Quantification of the reduced apparent elastic modulus *K* of dissociated brain cells (two-tailed Mann–Whitney test). Each point represents a single cell. **f**,**g**, Western blot quantification (*N* = 3; normalized to total protein) of NCAM1 (250 kDa) (**f**) and N-cadherin (**g**). **h**, AFM-based stiffness maps overlaid on bright-field images of control and NCAM1- and N-cadherin-depleted brains. Scale bar, 100 µm. **i**, Tissue stiffness quantification (nested *t*-test). Each point represents the median *K* of an embryo. **j**, Schematic illustrating the mechanism linking Piezo1 to tissue stiffness: Piezo1 regulates major cell–cell adhesion proteins (N-cadherin, NCAM1), which in turn regulate tissue stiffness. **k**, Western blot of Sema3A protein expression in control and NCAM1 and N-cadherin-depleted brains. **l**, Quantification of Sema3A protein expression (*N* = 3; normalized to total protein). **d**,**f**,**g**,**l**, show two-tailed ratio paired *t*-tests; *P-*values are indicated. In **b**,**e**,**i** boxes show first and third quartiles with median lines; whiskers show the spread of data. Double KD, optic tract and brain tissue depleted of Piezo1.

The mechanical properties of brain tissue depend on its building blocks and their connectivity^36^. To understand why tissue softens following Piezo1 downregulation, we first assessed whether Piezo1 knockdown had an effect on cell body densities in the tissue, which are a well-established contributor to tissue stiffness in the developing *Xenopus* brain^19,20^. In agreement with previous literature^19,20^, in control embryos the density of cells rostral to the optic tract was higher than the density caudal to it (Fig. 4a,b). Rostral and caudal cell densities were unaffected upon either exclusively downregulating Piezo1 in the optic tract or the developing brain, or downregulating Piezo1 in both the brain and the optic tract (*P*_rostral_ = 0.367 and *P*_caudal_ = 0.454, one-way analysis of variance) (Fig. 4b), indicating that the softening of the tissue following Piezo1 depletion could not be explained by changes in cell body densities.

**Extended Data Fig. 5:**
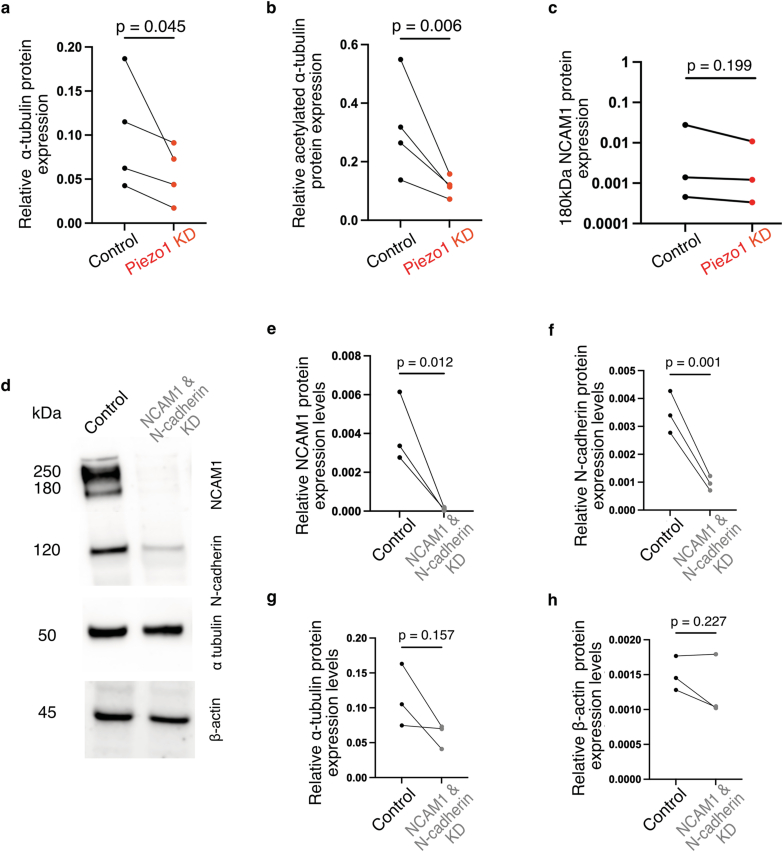
Cytoskeletal changes following genetic perturbations. **a-c**, Western blot quantification of **a**, α-tubulin, **b**, acetylated α-tubulin and **c**, 180 kDa NCAM1 for Western blot images shown in Fig. 4a. Data normalised by the total protein concentration; two-tailed ratio paired t-tests, *p-*values indicated. Each point represents the mean of a biological replicate (N = 4). **d**, Western blot images of NCAM1, N-cadherin, α-tubulin and β-actin expression in control and in NCAM1/N-cadherin double-knockdown samples (KD). **e-h**, Western blot quantification of morpholino-mediated knockdown of NCAM1 and N-cadherin for **e**, NCAM1, **f**, N-cadherin, **g**, α-tubulin, and **h**, β-actin. Data normalised by the total protein concentration; two-tailed ratio paired t-tests, *p-*values indicated. Each point represents the mean of a biological replicate (N = 3). KD: knockdown.

Because the cell densities did not change, we next assessed whether the mechanical properties of cells within the tissue were altered after Piezo1 knockdown. As cytoskeletal components are key contributors to cellular stiffness^37^, we first used Western Blots to analyse cytoskeletal protein expression in control and Piezo1-downregulated brains. The expression of β-actin was unaffected by Piezo1 downregulation (*P* = 0.410, ratio paired *t*-test) (Fig. 2k and Extended Data Fig. 3b), while α-tubulin levels decreased following Piezo1 downregulation (*P* = 0.045, ratio paired *t*-test) (Fig. 4c and Extended Data Fig. 5a). However, recent studies showed that the levels of microtubule acetylation rather than absolute α-tubulin levels affect cell mechanics^38,39^. In *Xenopus* brains, the ratio of acetylated to total α-tubulin was unchanged between Piezo1-downregulated and control brains (*P* = 0.165, ratio paired *t*-test) (Fig. 4d and Extended Data Fig. 5b), suggesting that the mechanical state of cells was not affected by Piezo1 knockdown.

To confirm if tissue softening was a consequence of cell softening, we measured the stiffness of acutely dissociated neuroepithelial cells from the developing brain using AFM. Single cell stiffness was not altered upon downregulating Piezo1 (*P* = 0.14, Mann–Whitney test) (Fig. 4e), indicating that cell-intrinsic mechanics did not contribute significantly to the decrease in brain tissue stiffness observed upon Piezo1 downregulation.

Having ruled out changes in cell densities and cellular stiffness as the cause of tissue softening due to Piezo1 depletion, we hypothesized that reduced cell–cell interactions might contribute to the observed softening. Western blot analysis was used to quantify protein expression levels of the two major cell–cell adhesion molecules found in the developing neuroepithelium: neural cell adhesion molecule 1 (NCAM1)^40^ and N-cadherin^41^. While the 180-kDa band of NCAM1, probably representing the unmodified protein^42^, showed no significant change (*P* = 0.199, ratio paired *t*-test) (Fig. 4c and Extended Data Fig. 5c), the 250-kDa polysialylated NCAM1^42^ band significantly decreased following Piezo1 depletion (*P* = 0.007, ratio paired *t*-test) (Fig. 4c,f). Additionally, N-cadherin expression significantly decreased following Piezo1 downregulation (*P* = 0.019) (Fig. 4c,g), indicating that Piezo1-mediated changes in cell–cell adhesion may contribute to the measured decrease in tissue stiffness (Fig. 3).

To substantiate this hypothesis, we downregulated both NCAM1 and N-cadherin by injecting translation-blocking morpholinos into both dorsal blastomeres of four-cell-stage embryos. Western blot analysis confirmed reduction in both NCAM1 (*P* = 0.012, ratio paired *t*-test) and N-cadherin protein expression (*P* = 0.001) (Extended Data Fig. 5d–f), but not of the α-tubulin (*P* = 0.157) or β-actin (*P* = 0.227) loading controls (Extended Data Fig. 5d,g,h). The downregulation of NCAM1 and N-cadherin led to a significant decrease in tissue stiffness (median *K*_control_ = 270 Pa, median *K*_KD_ = 184 Pa; *P* = 0.017, nested t-test) (Fig. 4h,i). Collectively, our findings thus provide strong evidence for Piezo1 modulating tissue stiffness not via changes in cell-intrinsic mechanics or cell density, but rather via regulating cell–cell adhesions (Fig. 4j).

Moreover, downregulating NCAM1 and N-cadherin resulted in a significant decrease in Sema3A protein expression (*P* = 0.010, ratio paired *t*-test) (Fig. 4k,l), indicating that tissue softening is indeed sufficient to decrease the expression of long-range chemical guidance cues.

## Environmental stiffness regulates guidance cue expression ex vivo

**Fig. 5:**
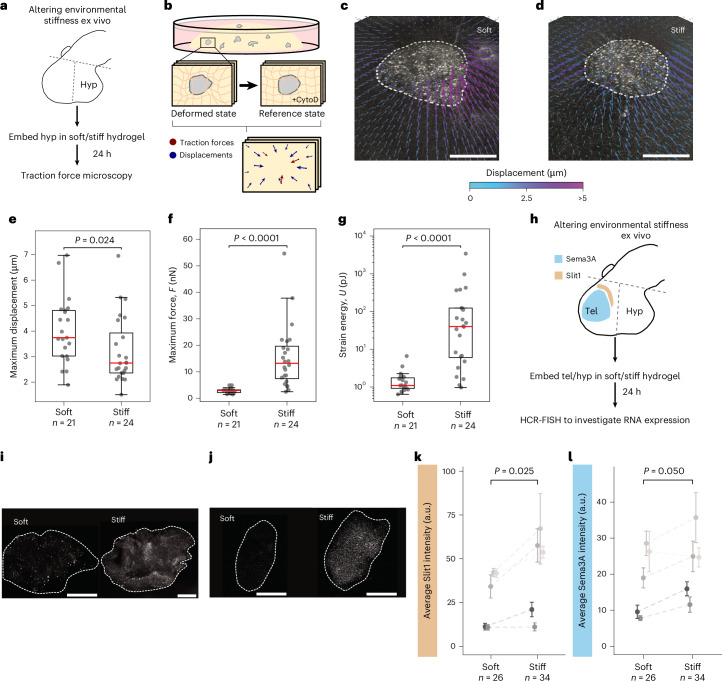
Altering environmental stiffness ex vivo affects traction forces and guidance cue expression. **a**,**b**, Schematic of the experimental design: hypothalamic (hyp) explants were dissected from *Xenopus* brains and embedded in collagen hydrogels for 24 h (**a**); the matrix was imaged in a deformed and a reference state (induced by tissue relaxation using cytochalasin D (CytoD)); deformations were quantified and forces calculated (**b**). **c**,**d**, Representative displacement fields for explants embedded in soft (**c**) and stiff (**d**) hydrogels. Displacements are colour-coded; white dashed lines indicate tissue boundaries. Scale bars, 100 µm. **e**, Maximum matrix displacement. **f**, Maximum traction force, *F*. **g**, The strain energy *U*, which is a measure of the work the tissue as a whole committed to deform the substrate. In **e**–**g**, each point represents an explant; boxes show first and third quartiles with median lines; whiskers extend to 1.5× the interquartile range (two-tailed Mann–Whitney *U*-test; *P* value indicated, *N* = 3). **h**, Schematic for perturbing environmental stiffness ex vivo. Telencephalon and hypothalamus (boundaries indicated in dashed lines) were embedded in soft or stiff 3D substrates. Slit1 and Sema3A mRNA expression were quantified by HCR-FISH after 24 h. **i**,**j**, Representative images of Slit1 (**i**) and Sema3A (**j**) expression in hypothalamic tissue in soft (left) and stiff (right) substrates. For guidance cue expression in the telencephalon, see Extended Data Fig. 6. Scale bars, 75 µm. **k**–**l**, Quantification of Slit1 (**k**) and Sema3A (**l**) mRNA expression. Points represent means ± standard errors of biological replicates (two-tailed ratio paired *t*-tests); *n* denotes number of tissue explants from five independent experiments. a.u., arbitrary units.

To test if stiffness could modulate chemical guidance cue expression independently of the decrease in Piezo1 expression, we exposed ex vivo brain tissue to different mechanical environments (Fig. 5a). We first quantified mechanical interactions between the tissue and its environment using 3D traction force microscopy^43^ (Fig. 5a,b). Tissue explants of wild-type stage 37/38 embryos—when axons already respond to Sema3A^18^ and Slit1—were embedded in soft (40 Pa) and stiff (450 Pa) 3D hydrogels (Fig. 5a,b)^43^ and matrix deformations quantified (Methods).

Cells adhered to the surrounding matrix, dynamically pulled on it and visibly deformed the matrix (Fig. 5c–e and Supplementary Video 1), thus probing its mechanical properties. 3D traction force microscopy revealed that the tissue exerted significantly larger forces *F* on the stiff hydrogels than on the soft ones (median maximum *F*_soft_ = 3 nN, *F*_stiff_ = 13 nN; *P* = 2 × 10^−7^, Mann–Whitney *U*-test) (Fig. 5f and Supplementary Video 2). These forces were probably exerted collectively by multiple cells in the explant^44^. In line with larger local forces in the stiff matrices, the total work done by the tissue to deform the matrix was also significantly enhanced in stiff environments (median strain energies *U*_soft_ = 1 pJ, *U*_stiff_ = 40 pJ; *P* = 1 × 10^−6^, Mann–Whitney *U*-test) (Fig. 5g). As the forces exerted by cells are experienced by their own intrinsic force sensors, such as Piezo1, the observed increase in traction forces in stiffer environments suggested an enhanced activation of mechanosensing proteins, potentially regulating the expression of Sema3A and Slit1.

**Extended Data Fig. 6:**
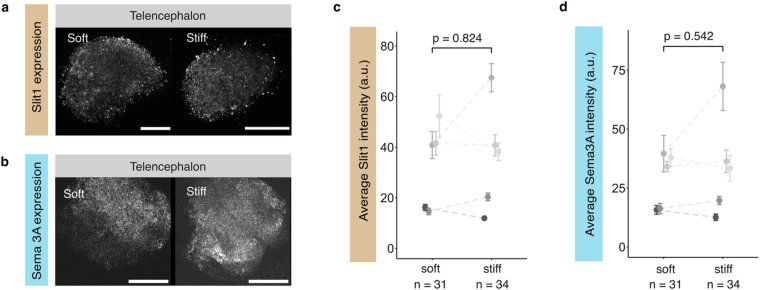
Chemical cue expression in the telencephalon is independent of substrate stiffness *ex vivo.* **a-b**, HCR-FISH images of **a**, Slit1 and **b**, Sema3A expression in telencephalic tissue after 24 hours in soft (left) and stiff (right) substrates (two-tailed ratio paired t-test, *p*-values indicated). Scale bars: 100 µm. **c-d**, Quantification of **c**, Slit1 and **d**, Sema3A mRNA expression. Individual points represent means with standard errors of each biological replicate. a.u.: arbitrary units. n denotes number of tissue explants from 5 independent experiments.

Thus, we next measured the effect of the environmental stiffness on Slit1 and Sema3A mRNA expression using HCR-FISH (Fig. 5h–l). Here, we used both stiff brain regions^19^ from the telencephalon and adjacent soft brain regions^19^ from the hypothalamus (Fig. 4a). After 24 h in culture, Slit1 and Sema3A expression in the stiffer telencephalic tissue—which typically produces the guidance cues in vivo—was unaffected by environmental stiffness (*p*_Slit1_ = 0.824, *p*_Sema3A_ = 0.542, ratio paired *t*-test) (Extended Data Fig. 6). However, in the softer hypothalamic tissue—which does not produce either guidance cue in vivo—we observed a significant increase in both Slit1 and Sema3A mRNA expression in stiff compared to soft substrates (*p*_Slit1_ = 0.025, *p*_Sema3A_ = 0.050, ratio paired *t*-test) (Fig. 5i–l). These data showed that altering environmental mechanics is sufficient to modulate the expression of chemical guidance cues in soft brain tissues ex vivo.

## Environmental stiffness regulates Sema3A expression in vivo

**Extended Data Fig. 7:**
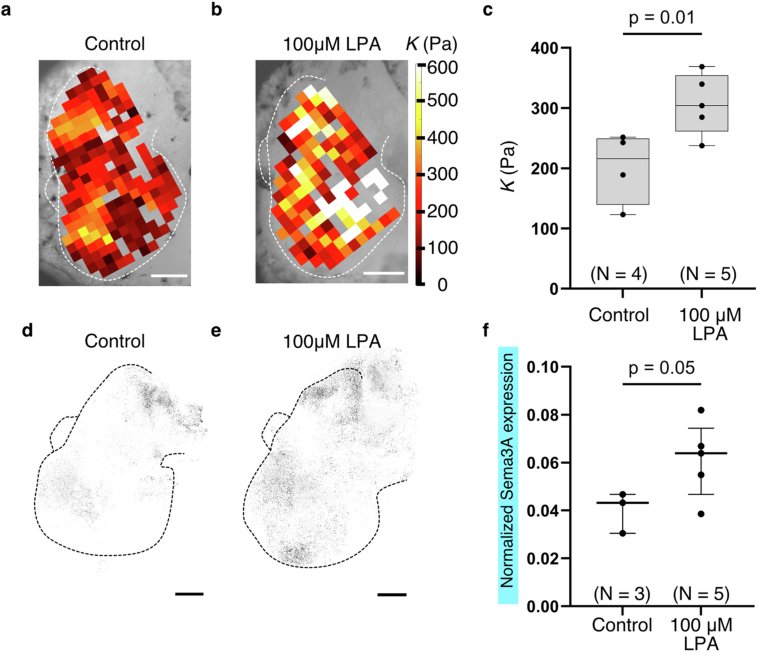
Lysophosphatidic acid (LPA) treatment results in increased tissue stiffness and Sema3A expression. **a-b**, Heatmaps of Stage 33/34 embryonic brains treated for 2-3 hours with **a**, 100 µM DMSO (control) or **b**, 100 µM LPA, overlaid on a brightfield image of the brain. The brain boundary is outlined by a white dotted line. Heat maps are scaled to an apparent elastic modulus *K* = 600 Pa. **c**, Median apparent elastic moduli *K* per animal in both conditions. Each dot in the graph corresponds to an animal measured. Boxes span the first and third quartiles with a line at the median, whiskers span the range of the data. Groups were compared with a nested t-test, where AFM measurements were nested under animals, and animals under condition (*p*-value indicated). **d-e**, Sema3A RNA expression in embryonic brains treated for 6 hours with **d**, 100 µM DMSO (control) or **e**, 100 µM LPA. The brain boundary is outlined by a black dotted line. **f**, Normalized Sema3A mRNA expression levels (two tailed unpaired t-test with Welch’s correction; *p-*value indicated). Each dot corresponds to an embryo measured. Scale bars: 100 µm. N: number of embryos measured. LPA: lysophosphatidic acid.

To corroborate the effect of tissue stiffness on the expression of long-range chemical signals in vivo, we first used a biochemical approach to increase the stiffness of brain tissue. We hypothesized that the application of lysophosphatidic acid (LPA), which enhances RhoA-mediated actomyosin-based contractility in neurons^45^, would increase tissue stiffness. Treatment of stage 33/34 embryos with 100 µM LPA for 2–3 h led to a significant increase in brain tissue stiffness (median *K*_control_ = 216 Pa, median *K*_LPA_ = 304 Pa; *P* = 0.01, Nested *t*-test) (Extended Data Fig. 7a–c). Consistent with the important role of tissue stiffness in regulating long-range chemical signal expression, Sema3A expression was significantly increased after 6 h of this treatment (*P* = 0.05, unpaired *t*-test with Welch’s correction) (Extended Data Fig. 7d–f).

To verify in vivo that Sema3A mRNA expression was indeed induced by the stiffening of the tissue and not due to some biochemical side effect from the LPA treatment, we then exploited the fact that the elastic modulus of brain tissue increases under uniaxial compression, thus effectively resulting in a stiffer environment^46^. To locally stiffen soft hypothalamic brain regions of stage 35/36 embryos, we used AFM to apply compressive forces of 30 nN for >6 h (until embryos reached stage 40) in vivo^19,47^.

**Extended Data Fig. 8:**
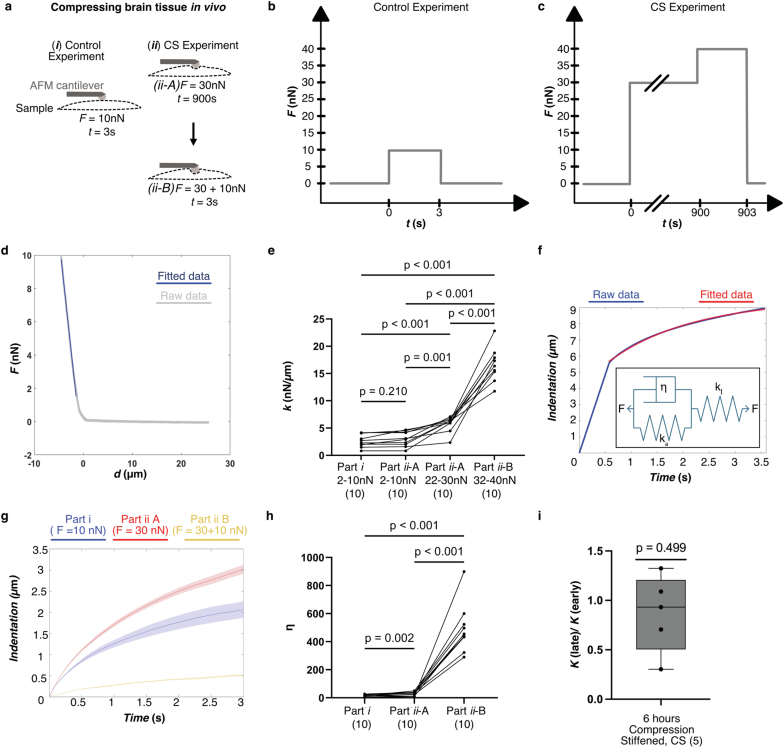
Uniaxial compression leads to brain tissue stiffening *in vivo.* **a-c**, Schematic of the experiment. In the experiment mimicking the control condition (**a**(i), **b**), the tissue was indented with *F* = 10 nN for a duration of *t* = 3 s. In the experiment mimicking the compression stiffening (CS) experiments (**a**(ii), **c**) (Fig. 6), the tissue was first exposed to a constant force of *F* = 30 nN for 900 seconds (ii-A), and subsequently, without retracting the cantilever, it was indented further by an additional *F* = 10 nN for 3 seconds (ii-B). **d**, Example of a linear fit (blue) to a force–distance (*F-d*) curve (grey) used to calculate the stiffness *k* (see Methods). **e**, Plot of the stiffnesses obtained by fitting *F = kd + c* through the last 8 nN of the *F-d* curves of indentations (i), (ii-A), and (ii-B), as well as through the range of 2 nN – 10 nN of indentation (ii-A). The stiffness of the tissue was similar during the first 10 nN indentation in approaches (i) and (ii-A), as expected. However, already during the application of the compressive force in approach (ii-A), tissue stiffness increased immediately. *k* was significantly smaller at the beginning (2-10 nN) of the indentation than at its end (22-30 nN indentation) (median *k*_2-10 nN_ = 3.0 nN/µm *vs. k*_22-30 nN_ = 6.2 nN/µm). When probing tissue stiffness after compression for 900 s (*ii-B*), *k* increased even further (median *k*_ii-B_ = 16.9 nN/µm). These data confirmed that uniaxial compression leads to tissue stiffening^18^ *in vivo*. As the developing *Xenopus* brain is fairly homogeneous at early stages of development^19^, an impact of potentially stiffer, deeper layers to the measured increase in stiffness it rather unlikely. **f**, Example indentation–time (*δ*-*t*) curve (blue) with a Standard Linear Model (SLM) fit (red); see Methods. Inset: schematic diagram of the SLM, which consists of a spring in series with an element containing a parallel dashpot (with viscosity *η*) and a second spring. **g**, Mean indentation–time (*δ*-*t*) curves for (i), (ii-A) and (ii-B), with the shaded regions representing +/- the Standard Error of the Mean. The creep (flow) of the tissue was relatively smaller for larger forces. **h**, Quantification of the apparent viscosity *η. η* increased significantly with increasing forces, indicating that the tissue became less fluid under compression, in particular on longer time scales. **e**, **h**, Data were compared with a repeated measures one-way ANOVA with Geisser-Greenhouse correction, followed by a Tukey’s multiple comparison test. Each dot represents an embryo measured (N = 10). **i**, The ratio of the median apparent elastic modulus *K* obtained in the last hour of the > 6 hour compression stiffening experiment to that obtained in the first hour was similar to 1 (one sample *t*-test), suggesting that tissue mechanics was only altered for as long as compression was applied. Each data point represents an embryo measured (N = 5). Numbers in parentheses indicate the number of measurements. The boxes span the first and third quartiles with a line at the median. The whiskers span the range of the data. All *p*-values are indicated in the figure. CS: compression stiffening.

Control measurements, in which a force of 10 nN was applied to embryonic brains with no prior compression (Extended Data Fig. 8a(i),b) and to the same brains after an initial indentation with a force of 30 nN for 15 min (mimicking the strain stiffening conditions) (Extended Data Fig. 8a(ii),c), confirmed that the stiffness *k* of the tissue immediately increased under compression (*P* = 0.001, repeated-measures analysis of variance (ANOVA) followed by Tukey’s multiple comparison) (Extended Data Fig. 8d,e), probably because of the non-linear elasticity of the tissue and water flow. Furthermore, using a standard linear model fit (Methods) to the indentation–time curves obtained above (Extended Data Fig. 8f), we found an increase in the tissue’s apparent viscosity *η* under compression (*P* < 0.001, repeated-measures ANOVA followed by Tukey’s multiple comparison) (Extended Data Fig. 8g,h), probably because of fluid exudation (loss of interstitial fluid), which increases internal friction and resistance to further deformation^48^. However, changes in the mechanical properties of tissue under compression were transient: the tissue’s apparent elastic moduli *K* were similar before and after compressing the brains for >6 h (*P* = 0.499, one-sample *t*-test) (Extended Data Fig. 8i).

**Fig. 6:**
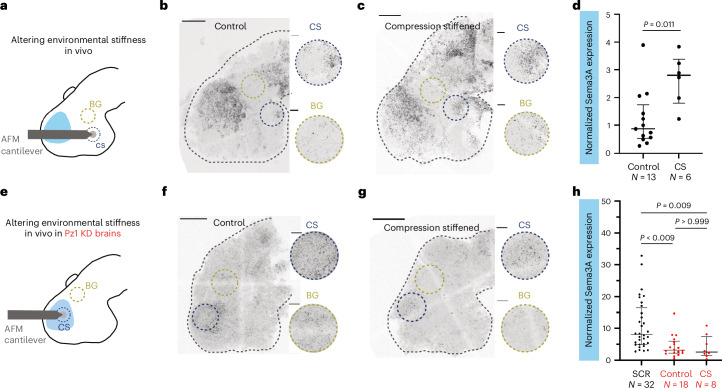
Stiffening brain tissue in vivo triggers Piezo1-dependent ectopic Sema3A expression. **a**, Schematic of the experimental set-up for locally increasing tissue stiffness in wild-type brains in vivo. The hypothalamus was compression-stiffened with an AFM probe for >6 h. **b**,**c**, Representative Sema3A HCR-FISH images of control (**b**) and compression-stiffened (**c**) brains. Insets: regions selected for analysis. **d**, Ratio of the total area covered by signal in the compression-stiffened (CS) region to the mean area covered by the signal in background (BG) regions were analysed (unpaired *t*-test with Welch’s correction; *P* value indicated). **e**, Schematic of the experimental set-up for locally increasing tissue stiffness in Piezo1 knockdown brains in vivo. The telencephalic region of the brain was compression-stiffened as in **a**. **f**,**g**, Representative Sema3A HCR-FISH images in control (**f**) and compression-stiffened (**g**) Piezo1 knockdown brains. Insets: regions selected for analysis. **h**, Ratio of the total area covered by signal in the CS region to the mean area covered by the signal in BG regions were analysed (Kruskal–Wallis test, *P* < 0.0001; Dunn’s post hoc test for multiple comparison; *P* values indicated). In **d**,**h**, each point represents an embryo; lower quartile, median and upper quartile are indicated by bars. Scale bars, 100 μm (whole brain), 20 μm (insets). SCR, scrambled control morpholino.

**Extended Data Fig. 9:**
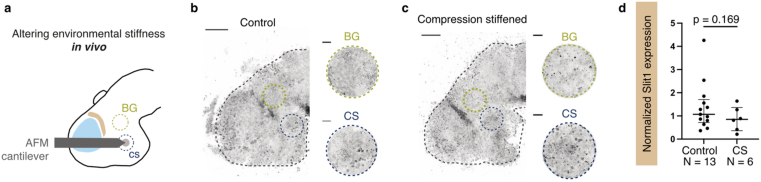
Compression-stiffening of hypothalamic tissue *in vivo* does not significantly alter Slit1 expression. **a**, Schematic representation of the experimental setup for compression stiffening brains *in vivo*. **b-c**, Slit1 expression in **b**, control and **c**, compression-stiffened brains. Dashed circles indicate compression-stiffened (CS) and background (BG) regions used for analysis. The insets show magnified view of analysis areas. Scale bars: 100 μm for whole brain images, 20 μm for insets. **d**, Normalised Slit1 expression levels (calculated by measuring the ratio of the total area covered by the signal in the CS region to the mean area covered by the signal in the BG regions). Individual dots represent the normalised expression levels in each embryo (N_control_ = 13 and N_CS_ = 6). The lines indicate interquartile ranges and the median. Two-tailed unpaired t-test with Welch’s correction was used for statistical analysis, the *p-*value is indicated. BG: background, CS: compression stiffened.

We then assessed mRNA expression in the compression-stiffened brains using HCR-FISH (Fig. 6a). Slit1 expression was not significantly different from controls in compression-stiffened brains (*P* = 0.169, unpaired *t*-test with Welch’s correction) (Extended Data Fig. 9). However, in line with our ex vivo data (Fig. 5l), stiffening the tissue resulted in the ectopic production of the chemical guidance cue Sema3A at the hypothalamus (Fig. 6c,d). In compression-stiffened brains, there was a significant increase in mRNA levels compared to controls (*P* = 0.011, unpaired *t*-test with Welch’s correction) (Fig. 6b–d), indicating that in vivo tissue stiffness may indeed regulate the expression of the long-range chemical signal, Sema3A.

Because Piezo1-depleted brains had decreased Sema3A expression (Fig. 2) and were softer than control brains (Fig. 3), and as an increase in tissue stiffness led to an increase in Sema3A expression in vivo (Fig. 5j,l), we finally tested if stiffening telencephalic regions, where Sema3A is normally produced, is sufficient to rescue Sema3A expression in Piezo1 knockdown brains (Fig. 6e). Compression-stiffening Piezo1-depleted stage 35/36 brains for >6 h did not restore Sema3A levels (*P*_control vs CS_ > 0.999; Kruskal–Wallis test followed by Dunn’s post hoc test) (Fig. 6f–h), suggesting that both tissue stiffness and adequate levels of Piezo1—which not only regulates but also detects tissue stiffness—are required to lay down the appropriate chemical landscape for supporting axon pathfinding in the developing brain.

## Outlook

During numerous processes in diverse biological systems, including brain development, cells encounter a plethora of chemical and mechanical cues in their environment, which they detect, integrate and interpret. We here discovered an intricate interplay between mechanical and long-range chemical signalling in vivo, with each influencing the other, demonstrating that neither cue can be fully understood in isolation. Importantly, local tissue stiffness regulates the availability of diffusive, long-range chemical signals, thereby impacting cell function at sites distant from the origin of mechanical signalling.

In tissues, cells constantly and dynamically exert forces on their environment. These contractile forces increase with increasing tissue stiffness (Fig. 5f). Larger forces, in turn, induce more augmented deformation of the cells’ intrinsic force sensors, such as Piezo1, thereby leading to their activation^49^. We found that Piezo1 serves at least two functions in the developing brain. First, it is involved in sensing tissue stiffness in both RGC axons^19^ (Fig. 1d,h) and the surrounding neuroepithelial cells (Figs. 1e and 6h). Second, it inherently contributes to regulating the mechanical properties of the tissue (Fig. 3a–d). The appropriate tissue stiffness in turn triggers the expression of long-range chemical guidance cues (Figs. 5 and 6).

Downregulation of Piezo1 in brain and skin tissues led to a decrease in tissue stiffness (Fig. 3 and Extended Data Fig. 4) and a subsequent decrease in the expression of Sema3A and Slit1 in the brain (Fig. 2). In contrast, the downregulation of Sema3A was followed by an increase in tissue stiffness (Fig. 3e–h), ruling out that the observed tissue softening following Piezo1 depletion was the direct consequence of decreased Sema3A expression.

The reason for the tissue stiffening following Sema3A depletion is currently unknown. Sema3A is known to decrease cell–extracellular matrix adhesion in various cell types, including neural crest cells^50^. Furthermore, Sema3A has been shown to decrease cell–cell adhesion via the internalization of cadherins^51^. Because our data show that cell junctions are an important regulator of tissue stiffness (Fig. 4), and Sema3A destabilizes cell junctions, Sema3A depletion might lead to the stabilization of cell junctions and thus to brain tissue stiffening. Regardless, our data show that feedback mechanisms between tissue mechanics and gene expression levels do not always act reciprocally.

Recent work has shown that environmental stiffness may regulate expression levels of Piezo1. For example, softer substrates lead to a decrease in Piezo1 expression levels in vitro^52,53^, Piezo1 levels in microglial cells correlate with the stiffness of Aβ plaque-associated brain tissues^54^, and in macrophages Piezo1 levels scale with the stiffness of ischaemic tissues^55^. We here found the opposite effect: a regulation of environmental stiffness by Piezo1 expression levels. A decrease in Piezo1 expression led to tissue softening in a developing vertebrate system in vivo (Fig. 3 and Extended Data Fig. 4), which was previously only observed in genetically induced *Drosophila* gliomas but not in non-transformed *Drosophila* brains^56^.

Although Piezo1 has been shown to regulate cell proliferation^24,57^ in other in vivo systems and the mechanical properties of epithelial cells^58^ in vitro, this was not observed here. Piezo1 depletion in the developing *Xenopus* brain in vivo had no effect on local cell densities or cell stiffness (Fig. 4a–e). Instead, Piezo1 downregulation resulted in a significant depletion of cell–cell adhesion (Fig. 4c,f,g), accompanied by a decrease in tissue stiffness and lower Sema3A levels (Figs. 2, 3 and 4h–l)—consistent with an important role of tissue stiffness in regulating the expression of long-range chemical signals. The simultaneous downregulation of Piezo1 and upregulation of NCAM1 and N-cadherin to rescue the phenotype in developing *X. laevis* brains was not feasible; however, the downregulation of these cell–cell adhesion molecules was sufficient to reduce tissue stiffness (Fig. 4h,i) and subsequently Sema3A expression (Fig. 4k,l), indicating that the mechanoregulation of tissue stiffness is not specific to Piezo1 but potentially mediated by NCAM1 and N-cadherin.

However, Piezo1 was required to induce Sema3A expression in response to tissue stiffness exceeding a critical threshold (Fig. 6h). While increasing tissue stiffness in vivo biochemically (Extended Data Fig. 7) or mechanically (Fig. 6) was sufficient to induce ectopic Sema3A expression in soft brain regions of wild-type embryos, tissue stiffening in Piezo-downregulated brains tissue failed to rescue Sema3A expression (Fig. 6), indicating that Piezo1 is required by the brain parenchyma to detect mechanical signals and induce Sema3A expression (Fig. 6h).

How Piezo1 activity contributes to regulating Sema3A expression remains to be studied. As Piezo1 is a non-selective cation channel that gives rise to calcium transients^26^, a regulation via calcium-dependent transcription factors such as activator protein (AP)-1^59^ or calcium-dependent enzymes such as mitogen-activated protein kinase/extracellular-signal-regulated kinase 1/2^59^, which are involved in the regulation of Sema3A expression^59^, is conceivable. Furthermore, softening tissue by depletion of Piezo1 or cell–cell adhesion proteins early in development may not only affect Piezo1-mediated mechanoresponses but potentially also neural stem/progenitor cell fate determination^25,60^ or FGF signalling^61^, which regulates the expression of both Sema3A and Slit1^17,35^. Future work will reveal the precise molecular details on how tissue mechanics and long-range chemical signalling impact each other.

A dependence of Sema3A expression on tissue stiffness is consistent with expression patterns found in healthy *Xenopus* brains. Normally, Sema3A is expressed in the telencephalon, which is significantly stiffer than the diencephalon and hypothalamus^19^, where Sema3A is not expressed (Fig. 2). Yet, Sema3A is already expressed in the telencephalon region at stage 32^35^—before the telencephalon stiffens^20^—indicating that other factors contribute to Sema3A regulation. Ex vivo, environmental stiffness affected Sema3A and Slit1 mRNA expression in the soft hypothalamus, which does not produce these guidance cues, but had no effect on the stiffer, guidance cue-producing telencephalic brain regions (Extended Data Fig. 6). These data suggested a sigmoidal relationship between tissue stiffness and the mechanical induction of guidance cue expression. Namely, at lower stiffness, expression remains low. However, upon reaching a certain critical stiffness threshold, chemical guidance cue expression is induced. Beyond this threshold, further increases in environmental stiffness have no additional effect on Sema3A expression levels. Hence, once the tissue is sufficiently stiff to produce the cue, further increasing environmental stiffness will not affect its expression.

Collectively, our data show that in the developing *Xenopus* brain, Piezo1 is critical for setting up the mechanical and, consequently, chemical landscape required for proper RGC axon guidance; it is involved in both RGC-intrinsic and cell-extrinsic regulations of axon pathfinding in the developing retinotectal system. Our findings raise the intriguing possibility that tissue mechanics may regulate the transcription of other diffusible long-range biochemical factors^62^ in various other organ systems—and vice versa. Through such bidirectional regulation, tissue mechanics could thus directly (through mechanotransduction pathways) and indirectly (through the regulation of chemical signalling pathways) impact cell function and tissue development over large distances. Understanding the interplay between chemical and mechanical signalling is challenging but has great potential to provide new insights into the regulation of development, physiology, ageing and disease.

## Methods

All reagents were obtained from Sigma-Aldrich unless otherwise specified.

### Animal experiments

#### Animal model

All animal experiments were approved by the Ethical Review Committee of the University of Cambridge, in compliance with guidelines set by the UK Home Office. Wild-type *X. laevis* embryos of both sexes were obtained via in vitro fertilization. Embryos were reared in 0.1 × Marc’s Modified Ringer’s Solution (MMR) or 0.1 × Modified Barth’s Saline (MBS) at 14–18 °C to reach the desired developmental stage, according to Nieuwkoop and Faber staging^33^. All animals used in this study were below embryonic stage 45.

#### Morpholino injection

Freshly fertilized embryos were chemically dejellied in 2% w/v cysteine in 0.1 × MMR/MBS, pH 8.0, for 2–5 min. Embryos were visually inspected under a stereomicroscope and when the jelly coat was no longer visible, embryos were rinsed in several washes with 0.1 × MMR/MBS. The dejellied embryos were gently transferred to a mesh-bottomed dish containing 4% w/v Ficoll in 0.1 × MMR/MBS, pH 7.5. Glass capillaries (1.0 mm outer diameter, 0.5 mm inner diameter; Harvard Apparatus) were pulled into needles and loaded with the morpholino construct. Needles were then affixed to a micromanipulator (connected to a FemtoJet 4X microinjector (Eppendorf)) and manually broken with forceps. The droplet size was calibrated in mineral oil, using a reticule in the microscope eyepiece, to dispense ∼5 nl of morpholino. Fluorescein- or lissamine-tagged translation-blocking morpholino oligonucleotides (MOs) targeting Piezo1 Tropicalis (5′-CACAGAGGACTTGCAGTTCCATCCC-3′)^19^, Piezo1.L- Laevis (5′-CGCACAGGACTTGCAGTTCCATCCC-3′)^39^, NCAM1 (5′-GATCCTTAATGTGCAGCATTGTAA-3′) and N-cadherin (5′- GAAGGGCTCTTTCCGGCACATGGTG-3′) were designed and synthesized by GeneTools. Then, 15 ng of either Piezo1 MO, NCAM1 or Control MO (GeneTools; 5′-CCTCTTACCTCAGTTACAATTTATA-3′) and 7.5 ng of N-cadherin MO were injected into the dorsal blastomeres of four-cell-stage embryos, as previously described^63^. For whole nervous system downregulation, both dorsal blastomeres were injected; for tissue-specific downregulation, one of the two dorsal blastomeres was injected. Embryos were reared until the requisite stage for use and, prior to downstream experiments, screened using a fluorescence stereomicroscope. Only embryos presenting a clear signal in the tissues of interest were selected.

#### Exposed brain preparation

Embryos were anaesthetized in *Xenopus* exposed brain medium (0.04% w/v tricaine methanesulfonate (MS222), 1 × penicillin–streptomycin–amphotericin (PSF) in 1.3 × MBS/MMR, pH 7.6)^64^; this higher osmolarity retards skin growth. Embryos were transferred to a Sylgard-coated Petri dish and immobilized laterally with bent 0.2-mm minutien pins (Austerlitz). Skin, dura and eye were dissected out using fine forceps and 0.1-mm/0.15-mm minutien pins (Austerlitz) in pinholders, to expose the brain from the dorsal to ventral midline and from the hindbrain to the telencephalon. Embryo viability throughout all in vivo experiments was assessed by the presence of a visible heartbeat, prior to processing the samples for downstream analysis.

#### LPA treatment

Stage 33/34 embryos were anaesthetized, and their brains were exposed as above and incubated for (1) 2–3 h for AFM-based stiffness mapping, and (2) 6 h for HCR-FISH, in exposed brain medium (0.04% w/v tricaine methanesulfonate (MS222), 1 × PSF in 1.3 × MBS/MMR, pH 7.6)^64^ containing either 100 μM DMSO (control) or 100 μM LPA (1-oleoyl-2-hydroxy-*sn*-glycero-3-phosphate; Avanti Research). Embryo viability was assessed by confirming the presence of a visible heartbeat and the samples were then processed for downstream analysis.

#### Brain-targeted electroporation

Finely controlled spatiotemporal knockdown of Sema3A was achieved using a targeted electroporation method^65^. The electroporation, rather than blastomere injection, approach was used for better spatiotemporal control of the downregulation and to avoid other developmental defects related to early Sema3A depletion^66^. Vitelline membranes were manually removed from stage 28 embryos using fine-tipped forceps. Stage 29/30 was chosen as axons had yet to grow across the contralateral brain surface, ensuring sufficient Sema3A depletion by the time the axons would encounter the cue.

Embryos were anaesthetized in (0.04% w/v tricaine methanesulfonate (MS222), 1 × penicillin–streptomycin–amphotericin B (PSF, Lonza, 1% v/v of a 100× solution containing: penicillin (10,000 U ml^−1^), streptomycin (10,000 μg ml^−1^), amphotericin B (25 μg ml^−1^)) in 1.0 × MBS, pH 7.6), and an individual embryo was placed ventral side down in a custom-fabricated Sylgard electroporation chamber with 0.5-mm-wide platinum electrodes on either side. Glass capillaries (1.0 mm outer diameter, 0.5 mm inner diameter) were loaded with 1 mM fluorescein-labelled validated Sema3A MO^34^ (GeneTools; 5′- TGCAATCCAGGTCAGAGAGCCCATG-3’) or standard control morpholino solution (GeneTools; 5′-CCTCTTACCTCAGTTACAATTTATA-3′) and placed in a micromanipulator connected to a picospritzer (Intracel). The fluorescein label of the constructs enabled the visualization of their localization.

The capillary was inserted between the eye and brain, such that the target region was between the positive electrode and capillary. Eight pulses of morpholino were dispensed, followed by eight 18-V shocks, for a duration of 50 ms, spaced 1 s apart using a TSS20 Ovodyne electroporator (Intracel). The electrodes were then removed, and the embryo was gently transferred with a Pasteur pipette to a dish containing 0.1 × MBS and left to develop at 20 °C to stage 40. Embryonic brains were exposed (‘Exposed brain preparation’) and screened for fluorescence at the telencephalon region of the brain. Embryos with signal at the region of the brain where Sema3A is normally expressed were selected for AFM measurements (Fig. 3e).

#### *X. laevis* whole brain immunohistochemistry

Four-cell-embryos were injected with Piezo1 MO in one of the two dorsal blastomeres. Stage 40 embryos were screened; the fluorescent side corresponded with MO-injected (and therefore Piezo1 depletion), whereas the non-fluorescent side had wild-type levels of Piezo1. Embryos were fixed in 4% PFA in 1 × PBS for 4 h at room temperature, followed by two washes in 1 × PBS. The brains were then dissected out in PBST solution (1 × PBS with 0.1% Triton X-100), using 0.1-mm/0.15-mm minutien pins (Austerlitz). The dissected brain samples were washed in PBST solution containing 0.2% BSA (bovine serum albumin) and blocked for 45 min at room temperature in blocking solution (PBST solution containing 0.2% BSA and 10% donkey serum). Samples were then incubated with rabbit anti-Piezo1 antibody (Proteintech, 28511-1-AP, 1:10 dilution in blocking solution) at 4 °C overnight. Samples were washed thrice in PBST solution for 30 min before adding the secondary antibody, anti-rabbit Alexa Fluor 594 (Abcam, ab150080, 1:300 dilution in blocking solution) for 2 h at room temperature. Samples were washed thrice in PBS and incubated with DAPI (1:10,000) in 1 × PBS for 15 min. The samples were then washed five times in 1 × PBS. Following this, the samples were upgraded serially, with 15-mi washes, in 30%, 50% and 70% glycerol. Samples were mounted, in 35-mm glass-bottomed dishes (Ibidi), laterally/dorsally as required. Both MO-injected and non-injected sides of the embryos were imaged (Leica SP8 confocal microscope; 40× oil; numerical aperture, 1.3; *z*-step size, 2 µm). Maximum intensity projections of the first 40 µm of each *z* stack was made in Fiji. The telencephalon was identified using the DAPI channel; the intensity of Piezo1 expression in the telencephalon was measured. Statistical analysis was done with GraphPad PRISM.

### Visualizing RGC axons

#### RGC axon labelling in vivo

Stage 40 embryos were fixed using 4% paraformaldehyde (PFA; Thermo Fisher Scientific) in 1 × phosphate-buffered solution (PBS; Thermo Fisher Scientific) for 2 h at room temperature or overnight at 4 °C. Post-fixation, embryos were rinsed in 1 × PBS and immobilized with bent 0.2-mm minutien pins (Austerlitz) on Sylgard-coated dishes. DiI (1,1′-dioctadecyl-3,3,3’,3′-tetramethylindocarbocyanine perchlorate, Molecular Probes) crystals were dissolved in 100% ethanol and heated for a few minutes at 65 °C. The DiI solution was then loaded into microinjection glass capillaries (1.0 mm outer diameter, 0.78 mm inner diameter; Harvard Apparatus) and connected to a FemtoJet 4X microinjector (Eppendorf). DiI was injected into the eye, between the boundary of the lens and retina^67^, until a full roseate ring forms. After 24–28 h in 1 × PBS at room temperature, the brains were dissected and mounted in 1 × PBS in lateral view. *z*-stack images were obtained using a confocal microscope (SP8, Leica Microsystems; 20× air; numerical aperture, 0.75; *z*-step size, 1 μm).

#### RGC axon phenotype analysis

Only samples that were mounted with the brain positioned properly and with visible tracts were used in the phenotype and elongation analysis (below). DiI-labelled images were randomized and optic tracts with normal projections (axons reach the tectum, without mid-optic tract bend defects or visibly straying axons), or that displayed defects such as mid-optic tract bend stalling, or axon misprojections were counted. Misprojections frequently occurred near the mid-optic tract bend but also included axons straying in other parts of the tract. Some of the misprojections included deviating at the mid-optic tract bend, bypassing the bend, avoiding the tectum or projecting into the telencephalon. Percentages of each phenotype were compared for the various experimental conditions.

#### Optic tract elongation analysis

Maximum projections were made across 10- to 15-μm confocal image stacks of wholemount brains with DiI-labelled optic tracts. The optic tracts were manually outlined in Adobe Illustrator. Elongation of the optic tract was calculated by the major-to-minor axis ratio using a automated MATLAB algorithm^19^. Briefly, the major and minor axes were determined by fitting ellipses, with the same normalized second central moment as the optic tract area, around the optic tracts.

### Visualizing mRNA expression in situ

#### In situ hybridization

Whole-mount in situ hybridization was adapted from refs. ^18,68^. Stage 40 embryos were collected in cold PBS and fixed with 4% PFA (Thermo Fisher Scientific) for 2 h at room temperature. Whole brains were carefully dissected out in nuclease-free PBS (Thermo Fisher Scientific) and washed thrice with cold PTw (0.1% Tween 20 in RNase-free PBS). Samples were transferred to a 12-well plate with mesh adapters and treated with 20 μg ml^−1^ of proteinase K for 5 min at room temperature, then washed twice for 5 min in freshly prepared 2 mg ml^−1^ glycine in PTw. Brains were postfixed in 0.2% glutaraldehyde in PBS for 20 min at room temperature. Samples were washed thrice for 5 min in PTw. After treatment with freshly prepared 0.1% sodium borohydride in PTw for 20 min, brains were washed a further three times in PTw, and twice in hybridization buffer (50% formamide (Thermo Fisher Scientific), 750 mM NaCl, 1 × PE (10 mM PIPES, pH 6.8; 1 mM EDTA), 100 μg ml^−1^ yeast t-RNA (Thermo Fisher Scientific), 0.05% heparin and 1% SDS in nuclease-free water) for 5 min at room temperature. Samples were prehybridized for at least 1 h at 63 °C in a hybridization oven, in a humidity chamber, and samples were hybridized with 2 μg ml^−1^ of DIG-labelled Sema3A probe in hybridization solution overnight at 63 °C. A pCS2 ± *XenopusSema3A*^34^ plasmid, from the laboratory of C. Holt, was used as a template to prepare the DIG-labelled RNA probe. Samples were then washed in hybridization buffer at 63 °C for 30 min, and twice for 30 min with 300 mM NaCl, 1 × PE and 1% SDS at 63 °C. Followed by two 30-min washes in 50 mM NaCl, 1 × PE and 0.1% SDS at 50 °C, and a brief rinse in NTE buffer (500 mM NaCl, 10 mM Tris–HCl, pH 8.0; 1 mM EDTA). The samples were then treated with 100 μg ml^−1^ RNaseA (Thermo Fisher Scientific) and 100 U ml^−1^ of RNaseT1 in NTE for 60 min at 37 °C and rinsed briefly in NTE. The samples were then washed in 50% formamide, 300 mM NaCl, 1 × PE, 1% SDS at 50 °C for 30 min, followed by 50% formamide, 150 mM NaCl, 1 × PE, 0.1% Tween 20 at 50 °C for 30 min. Finally, the samples were washed twice at room temperature, and once for 20 min with 500 mM NaCl, 1 × PE, 0.1% Tween 20 at 70 °C to inactivate endogenous alkaline phosphatases. Samples were blocked in 1 × MABT (0.1 M maleic acid, 150 mM NaCl, 0.1% Tween 20, pH 7.5), with 2 mM levamisole (Abcam), 2% Boehringer blocking reagent (Roche), and subsequently incubated overnight at 4 °C with a 1:5,000 dilution of anti-digoxigenin antibody (Roche; 11093274910) in blocking reagent. After thorough washings, thrice quickly and 5 or 6 times for 1 h each with freshly made 2 mM levamisole in 1 × MABT, samples were washed twice for 20 min with 2 mM levamisole in NTMT (100 mM NaCl, 100 mM Tris, pH 9.5, 50 mM MgCl_2_, 0.1% Tween 20). The colour reaction was initiated by removing the NTMT and adding BM Purple (Roche) to the brains and stopped with the changes of PTw containing 1 mM EDTA. Samples were mounted laterally in PBS and imaged using a Leica MZFLIII stereomicroscope with QImaging microPublished Color RTV camera, and QCapture Pro 7 software.

#### Whole brain and in vitro tissue culture HCR-FISH

To visualize RNA in fixed *Xenopus* tissue, a modified HCR RNA-FISH protocol^69^ was used. Embryos/embedded tissues were fixed in modified Carnoy’s fixative (6 parts 100% ethanol, 3 parts 37% formaldehyde (Fisher Chemical) and 1 part glacial acetic acid (Fisher Chemical))^70^ for 4 h at room temperature or overnight at 4 °C. Samples were rinsed in 70% ethanol in PBST (PBS + 0.1% Tween 20) for 10 min and dehydrated by three 30-min washes with 100% ethanol. Thereafter, samples were stored at −20 °C and then rehydrated serially with 70%, 50% and 25% ethanol in PBST for 15 min each. Samples were then rinsed in PBST thrice for 15 min. For embryos, samples were kept in PBST and brains were dissected out with 0.1-mm/0.15-mm minutien pins (Austerlitz) in pinholders. For embedded tissues, the samples were dissected out of the 3D gel matrix using 0.1-mm/0.15-mm minutien pins (Austerlitz) in pinholders. The dissected brains/tissues were transferred to a four-well plate and bleached in a solution of 5% formamide, 2.5% 20 × SSC (Promega) and 28% H_2_O_2_ for 45 min under a Zeiss Cold Light Source at 100% intensity. The samples were gently rinsed and then washed thrice in PBST for 10 min each. They were then treated for 10 min at room temperature with 5 mg ml^−1^ proteinase K diluted in PBST, followed by three 5-min washes with PBST. They were fixed again with a 20-min wash with 3.7% formaldehyde in PBST and washed five times for 5 min each in PBST.

The samples were washed in 1 ml of probe wash buffer (Molecular instruments) (preheated to 37 °C) for 5 min at room temperature and then washed in 500 ml of hybridization buffer for 30 min at 37 °C. The probe solution was prepared by mixing custom probes (Molecular instruments) in 500 ml of hybridization buffer (Molecular instruments). The final concentrations were 24 nM for Sema3A and 12 nM for Slit1. The probe mixture was heated at 37 °C for 30 min. The probe solution was then added to the samples and incubated overnight at 37 °C. Samples were kept stationary. The following day, the wash buffer was preheated to 37 °C in a water bath. Excess solution was removed, and the samples were washed twice for 30 min each in 1 ml of preheated wash buffer at room temperature. The samples were then washed for 5 min in 50% 5 × SSCT (5 × SSC (diluted in PBS) + 0.1% Tween 20) + 50% wash buffer at room temperature, following which they were washed with 5 × SSCT, two times for 20 min each, at room temperature. Thereafter, they were preamplified with 1 ml of amplification buffer (Molecular instruments) for 10 min. Then, 4 μl of H1 and 4 μl H2 hairpins, from 3 μM hairpin stocks (Molecular instruments) were taken and separately heated at 95 °C for 90 s and cooled for 30 min at room temperature in the dark, after which 8 μl of the prepared hairpin solution was mixed with 500 μl amplification buffer. The final concentration of the hairpins was 48 nM. Excess amplification buffer from the samples was removed and the hairpin mix was added and incubated at room temperature, overnight, in the dark. The samples were washed twice with 5 × SSCT, 30 min each in 1 × PBS. They were then incubated with DAPI 1:10,000 solution in PBS for 10 min and washed twice with 1 × PBS for 10 min each. The samples were then serially upgraded with 15-min washes until samples sunk to the bottom of the dish in 30%, 50% then 70% glycerol in PBS. Samples were stored at 4 °C in 70% glycerol. The samples were mounted (laterally for whole brains) in 70% glycerol and 80-μm *z* stacks were acquired on a laser scanning confocal microscope (SP8, Leica Microsystems; 40× oil; numerical aperture, 1.3; *z*-step size, 2 μm). For experiments involving LPA treatment, brains were imaged on an inverted fluorescence light microscope (Thunder DMi8, Leica Microsystems; 63× oil; numerical aperture, 1.4; *z*-step size, 1 μm). Multiple tiles were acquired for each sample, and tile stitching was performed, using Leica LAS X software.

#### HCR-FISH quantification

To quantify Slit1 expression in whole brains, a sum intensity projection was obtained for 30 μm from the *z* stack where DAPI first appears. This sum intensity projection was used for further analysis. A rectangular ROI covering the Slit1-producing region at the boundary of the diencephalon and telencephalon was selected. The area covered by puncta was calculated using the analyse particles plugin in Fiji^71^. A ratio of the total area covered by the puncta in the Slit1-producing region to the total area of the forebrain (diencephalon, telencephalon and hypothalamus) was calculated and this value used for statistical analysis.

For the quantification of Sema3A expression in whole brains, a maximum intensity projection was obtained for 60 μm from the *z* stack where Slit1 first appears (because Sema3A expression is ventral to Slit1). This maximum intensity projection was used for further analysis. Circular regions of interest (ROIs) were selected in proportion to the size of the brain. One ROI was selected, and one or more background regions were selected in each brain. The ROIs and background regions were selected using the DAPI channel, with ROI being the Sema3A-producing region (telencephalon) and background being non-Sema3A-producing regions (that is, the diencephalon and hypothalamus), respectively. The images were manually thresholded (ensuring all RNA puncta were efficiently segmented and the threshold limits were set at the same value for all the images within a replicate) and a binary image was created to segment the RNA puncta. The area covered by the signal was found using the analyse particles plugin in Fiji^71^. Where more than one background region was used, the average area covered by the punctae were used.

$${\mathrm{Normalized}}\,{\mathrm{expression}}=\frac{{\mathrm{Total}}\,{\mathrm{area}}\,{\mathrm{covered}}\,{\mathrm{by}}\,{\mathrm{punctae}}\,{\mathrm{in}}\,{\mathrm{ROI}}}{{\mathrm{Average}}\,{\mathrm{area}}\,{\mathrm{covered}}\,{\mathrm{by}}\,{\mathrm{punctae}}\,{\mathrm{in}}\,{\mathrm{background}}}$$

This normalized expression value was used for further statistical analysis. Data were assessed with an unpaired *t*-test with Welch’s correction for unequal standard deviation using GraphPad Prism.

To quantify Sema3A expression in 100 µM LPA-treated embryos, the images were computationally cleared using the Thunder settings in the Leica LAS X software (mounting medium, glycerol; refractive index, 1.47290). Thereafter, a maximum intensity projection was obtained for the first 30 μm. The DAPI channel was used to outline the whole brain including the diencephalon, telencephalon, hypothalamus and tectum. Thereafter, the images were thresholded and analysed similarly as described above. The area covered by Sema3A punctae was calculated for the whole brain. Normalized expression was calculated by taking a ratio of the area covered by Sema3A punctae to the total brain area selected. This normalized expression value was used for statistical analysis. Data were compared using an unpaired *t*-test with Welch’s correction for unequal standard deviation using GraphPad Prism.

To quantify the mRNA expression of ex vivo brain regions embedded in hydrogels, the images obtained were randomized and a number was assigned to each image, for blinded analysis. Using the DAPI channel, the tissue regions were first segmented separately for the first 40 μm of the image stacks (that is, 20 *z* slices) using a custom-written Fiji script. Additionally, a circular background ROI (5% of image dimensions) was selected where no tissue or collagen hydrogel was present. Mean intensity measurements were then performed in the Sema3A and Slit1 channels for both areas. Subsequently, a median value of the background subtracted signal of interest was calculated in each dissected tissue from all the optical slices. To compare between soft and stiff conditions, mean values were derived independently for each group. Data were assessed using GraphPad Prism.

For HCR-FISH analysis of the compression stiffening experiments, an image of the embryo was taken after compression stiffening using a charge-coupled device camera (Imaging Source) mounted on a TopViewOptics upright imaging system (JPK Instruments). An outline was drawn around the brain, specifically the optic tectum and the bead glued to the cantilever. This was overlayed in Affinity software with the bright-field image of the brain obtained from confocal imaging of the sample with an Sp8 Leica Microsystems laser scanning confocal microscope to identify the precise region of compression. The region of compression was taken as the ROI and one to two background (BG; non-compressed) regions in the hypothalamus and the diencephalon (caudal to Slit1 expression at the boundary of the telencephalon and diencephalon) were selected. A maximum intensity projection of the first 40 μm was created for both Sema3A and Slit1, and thereafter the images were manually thresholded and segmented, and normalized expression levels were calculated by the same method as used for the Sema3A expression in whole brain section.

#### Western blotting

Brains of stage 39/40 *Xenopus* embryos were dissected from scrambled morpholino (controls) and Piezo1 morpholino-injected embryos. The dissected tissue was mechanically homogenized in 100 µl RIPA lysis buffer (NaCl 150 mM, Triton X-100 1%, sodium deoxycholate 0.5%, sodium dodecyl sulfate 4 mg ml^−1^, Tris buffer 50 mM, pH 8, 1× halt protease and phosphatase inhibitor cocktail (Thermo Scientific)), by pipetting 50 times and protein samples were prepared for Western blots^72^. Samples were vortexed for 30 s and then centrifuged at 17,900*g* for 15 min at 4 °C. The supernatant was taken and its protein concentration was measured using the Bradford assay with bovine serum albumin to generate a standard curve. Samples where diluted with RIPA lysis buffer to ensure a final concentration of 1 mg ml^−1^ after adding sample buffer (Nupage NP0007)/and 5% v/v β-mercaptoethanol. Samples were denatured on a heat block for 5 min at 95 °C and kept on ice until loading 20 µl of each sample on to precast 4–12% Bis-Tris NuPage gradient gels (Invitrogen). Thereafter, samples were transferred to a nitrocellulose membrane (Biorad). Membranes were blocked for 30 min in a blocking solution of 5% skim milk powder diluted in Tris-buffered saline (TBS, pH 7.6), and incubated for 1 h at room temperature with a polyclonal rabbit anti-Piezo1 (anti-FAM38A) primary antibody (NBP1-78446; 1:1,000 dilution), polyclonal rabbit anti-Sema3A antibody (ab199475; 1:500 dilution), monoclonal mouse anti-β-actin antibody (ab6276; 1:1,000 dilution), monoclonal mouse anti-NCAM1 (DSHB catalogue number 4d, RRID:AB_528389; 1:2,000 dilution), polyclonal sheep anti-N-cadherin (AF6426-SP; 1:500 dilution), monoclonal rabbit anti-α-tubulin (acetyl K40; ab179484; 1:1,000 dilution), monoclonal mouse anti-α tubulin (ab7291; 1:1,000) in a primary antibody solution of 5% skim milk powder diluted in TBST (TBS + 0.05% Tween 20, pH 7.6). Primary antibodies were then washed off with TBST and the nitrocellulose membrane was incubated for 1 h at room temperature(18–22 °C) in polyclonal goat anti-rabbit antibody conjugated to horseradish peroxidase (HRP) (ab97080; 1:10,000 dilution) for Piezo1, Sema3A and anti-α-tubulin (acetyl K40), a polyclonal donkey anti-sheep antibody conjugated to HRP (HAF016; 1:1000 dilution) for N-cadherin, and a polyclonal goat anti-mouse antibody conjugated to HRP (ab6789; 1:10,000 dilution) secondary for β-actin, NCAM1, α-tubulin, in a secondary antibody solution of 5% skim milk powder diluted in TBST (pH 7.6). Western blots were developed using Super Signal West Femto Maximum Sensitivity Substrate ECL (Thermo Fisher Scientific) and the Li-Cor Odyssey FC imaging system. Signal intensities were measured using Li-Cor Image Studio Lite software. The ratio of relative intensities of different proteins to total protein staining (Revert 700 total protein stain and wash solution) was used to compare different groups. The average intensities of each protein normalized to total protein stain was calculated for each experiment. To quantify the relative proportion of acetylated α-tubulin in the lysates, the ratio of the relative expression of acetylated α-tubulin to total α-tubulin was obtained.

#### Cell density visualization and analysis

Whole-mount stage 40 *Xenopus* brains with DiI-labelled optic tracts were incubated in 1 μg ml^−1^ DAPI (4′,6-diamidino-2-phenylindole, Santa Cruz Biotechnology) diluted in 1 × PBS for 10 min. Brains were washed thrice in 1 × PBS for 10 min and laterally mounted in 1 × PBS for imaging. *z* stacks were acquired using an SP8 confocal microscope (Leica Microsystems; 20× air; numerical aperture, 0.75; *z*-step size, 1 μm). Image stacks were imported into Fiji. For each brain, the image at which the mid-OT bend was in focus was selected. A maximum intensity projection of this image along with the optical section above and below were made. Two 50 μm × 50 μm ROIs were selected, rostral and caudal to the optic tract bend, and a Gaussian blur filter (sigma, 2.0) was applied. Images were manually thresholded to capture all nuclei as accurately as possible. Images were binarized and the ‘Analyse Particle’ function (size, 1–∞; circularity, 0.2–1.00) was used to acquire the area occupied by nuclei in each region. Relative nuclear density was taken as the ratio of the area occupied by nuclei to the total area of the ROI.

### AFM

#### Stiffness mapping

Tipless silicon cantilevers (Arrow TL1, NanoWorld) were calibrated using the thermal noise method^73^ to determine the spring constant *k*, and those with *k* between 0.02 and 0.04 N m^−1^ were selected. Monodisperse spherical polystyrene beads (diameter, 37.28 ± 0.34 µm; microParticles) were glued to the cantilever ends as probes with M-Bond 610 (Agar Scientific). Cantilevers were mounted on a CellHesion 200 AFM head (JPK Instruments), which was set up on an *x*/*y* motorised stage (JPK Instruments) controlled by custom-written Python scripts^19,74^. Stage 40 embryos (controls/Piezo1 knockdown/Sema3A knockdown/NCAM1 and N-cadherin knockdown) or stage 35/36 embryos (LPA treated) were anaesthetized with one hemisphere of the brain exposed (‘Exposed brain preparation’) and transferred to a Petri dish on the motorized stage. Indentation measurements were performed automatically in a user-defined grid with a maximum indentation force of 10 nN, an approach speed of 5 µm s^−1^ (20 µm s^−1^ for LPA treated and NCAM1/N-cadherin knockdown embryos), at a data rate of 1,000 Hz (2,000 Hz for NCAM1/N-cadherin knockdown embryos). After each measurement, the cantilever was retracted by 100 µm, and the stage moved by a set distance (20–25 µm) to the next position^74,19^. For skin stiffness measurements (controls/Piezo1 knockdown), stage 28–31 embryos were anaesthetized (0.04% w/v tricaine methanesulfonate (MS222), 1.3 × MMR, pH 7.6) and immobilized on Sylgard-coated dishes (40 mm, TPP). Bright-field and GFP fluorescence overview images were obtained at 25× and 56× magnification with a stereofluorescence microscope (AxioZoom.V16, Zeiss) equipped with a Zyla sCMOS camera (Andor) mounted over the AFM set-up. Using a custom-written MATLAB script, a grid of AFM measurement points (40 µm × 40 µm) was defined on the skin overlaying the eye primordium, based on the GFP fluorescence of the injected control (scrambled MO)/Piezo1 MO, typically resulting in 21 measurements. Indentation measurements (force setpoint, 2 nN; approach speed, 20 µm s^−1^; data rate, 2,000 Hz).

#### Quantification of the reduced apparent elastic modulus *K*

Force–distance curves obtained from stiffness measurements were analysed with a custom-written MATLAB script^19,74^ to obtain the reduced apparent elastic modulus *K*. Raw AFM data were fitted to the Hertz model:

$$F=\frac{4}{3}K{R}^{1/2}{\delta }^{3/2}$$

where *F* is the applied force, *K* is the reduced apparent elastic modulus *E*/(1 − *v*^2^) (where *E* is the Young’s modulus and *v* is the Poisson’s ratio), *R* is the radius of the indenter and *δ* is the indentation depth^75,76^.

For exposed brain measurement, force–distance curves were analysed at the maximum applied force *F* = 10 nN. Points where the AFM data were not analysable were excluded. Criteria for excluding individual force–distance curves were (1) inability to apply linear fits through the baseline region, for example, due to noise; and (2) inability to apply good-quality Hertzian fits to the indentation region, that is, when the fit aligned poorly with the raw data. The grid of *K* values was plotted as heatmaps in MATLAB; non-analysable measurements were mapped in dark grey. When brain stiffness was calculated by region, a MATLAB script^19^ was used to manually select ROIs using anatomical boundaries and fluorescence labelling of structures as an approximate guide. For exposed brain measurements, ROI areas were selected four times and pixels defined as part of the ROI if selected at least three out of the four times. For skin measurements, force–distance curves were analysed at the maximum applied force *F*. Further to the criteria described above, force–distance curves were excluded from analysis if the maximum applied force deviated by more than 10% from the force setpoint of 2 nN. Scripts used for AFM measurements on skin are available from ref. ^77^. To determine if the elastic modulus changes during six hour compression stiffening (CS), we compared the median modulus from three force–distance curves acquired in the first hour with the median modulus from three force–distance curves acquired in the last hour. The ratio of the median elastic moduli obtained during both time points was calculated for each embryo.

#### Compression stiffening

To induce sustained local compression stiffening^19^, tipless silicon cantilevers with *k* > 0.1 N m^−1^ were selected (NanoWorld Arrow TL1 AFM probe) and polystyrene beads of 89.3–123 μm diameter (microParticles) were attached and used to apply a constant force of 30–40 nN in anaesthetized and exposed stage 35/36 embryos (‘Exposed brain preparation’) where (1) the hypothalamus of wild-type embryos or (2) the telencephalon of Piezo1-downregulated embryos were compressed. The force was applied for >6 h at 20–25 °C. Uncompressed controls were treated in the same way except for the AFM application. After every 5–20 min of compression, the cantilever was lifted briefly (<10 s) to allow the software to adjust for any drift on the AFM photodiode and a force–distance curve was saved. After removal of the cantilever after >6 h, compression-stiffened and control embryos were fixed in modified Carnoy’s fixative within 5 min and processed for HCR-FISH.

To verify stiffening of brain tissue under uniaxial compression^46^ and also to investigate potential changes in tissue viscosity, AFM-based creep measurements were conducted twice on each embryo (Extended Data Fig. 8a). Measurements were carried out with spherical probes of 44.65 µm radius (microParticles) glued to a SICON-TL cantilever (Applied NanoStructures). Spring constants were determined before the addition of the bead using the thermal noise method implemented in JPK software and were found to be ∼0.18 N m^−1^.

First, indentation measurements were conducted with an extend speed of 10 µm s^−1^ and a force setpoint of 10 nN. After reaching the setpoint force *F* = 10 nN, this force was maintained for 3 s, and the tissue’s creep response was recorded. Finally, the cantilever was retracted (Extended Data Fig. 8a(left),b, control experiment (i)). Subsequently, we conducted compression stiffening experiments by applying a force *F* = 30 nN, which was maintained for 900 s (Extended Data Fig. 8a(right),c, CS experiment part (iiA)). At the end of this force clamp (that is, the end of the compression stiffening experiment), the cantilever was moved downward by an additional 10 nN, and another force-hold of 3 s at *F* = 30 nN + 10 nN = 40 nN was performed (Extended Data Fig. 8a(right),c, CS experiment part (iiB)) to assess tissue stiffness at the end of the compression stiffening experiment.

Because of the lack of a baseline in experiment (iiB), data could not be fitted with the Hertz model to extract an apparent elastic modulus, *K* (in Pa, N m^−^^2^), as in standard indentation measurements. Instead, we fitted a linear function *F* *=* *kd* + *c* to the initial slopes of the force (*F*)–distance (*d*) curves in approaches (i), (iiA) and (iiB) to extract a stiffness *k* (in nN µm^−1^) (Extended Data Fig. 8d,e). The stiffness *k* scales linearly with the apparent elastic modulus *K* over a wide range of measurement parameters^74^. In all three approaches, the last 8 nN of the extended segments (before the start of the force clamp) were fitted; in approach (iiA) the initial part of the slope, between 2 and 10 nN, was also fitted.

To assess the viscosity of the tissue, we fitted a standard linear model, consisting of a spring (with spring constant *k*_l_) in series with an element containing a dashpot (with viscosity *η*) and a second spring (with spring constant *k*_a_) in parallel (Extended Data Fig. 8f, inset) to the indentation–time data. Here, the indentation *δ* at time *t* is given as

$$\delta \left(t\right)={\,\left[\frac{3}{4}\frac{{F}_{{\rm{c}}}}{\sqrt{r}}{\alpha }^{1-\alpha }{\Delta t}_{{\rm{a}}}^{\alpha -1}\left({C}_{0}+\left({C}_{0}-{C}_{1}\right){e}^{-t{C}_{2}}{\atop{2}}F_{1}\left(\alpha ,\alpha +1,\Delta {t}_{{\rm{a}}}{C}_{2}\right)\right)\right]}^{2/3}$$

where

$${C}_{0}=\frac{1}{{k}_{{\rm{l}}}+{k}_{{\rm{a}}}},\,\,{C}_{1}=\frac{1}{{k}_{{\rm{l}}}},\,\,{C}_{2}=\frac{{k}_{{\rm{l}}}{k}_{{\rm{a}}}}{\eta \left({k}_{{\rm{l}}}+{k}_{{\rm{a}}}\right)}$$

and *r* is the radius of the indenter, Δ*t*_a_ is the time taken to ramp the force up to the clamp force *F*_c_, *α* is a free parameter describing the shape of the force ramp (1 < *α* < 2), and _2_*F*_1_ is the hypergeometric function. This equation was fitted to the first 3 s of the creep data, that is, the indentation *δ* versus time *t*, during the force-hold using a custom-written MATLAB script^78^.

### In vitro techniques

#### Single cell dissociation for AFM stiffness measurements

*Xenopus* embryos at stage 39–40 were anaesthetized in MS222 solution (0.04% w/v tricanemethanesulfonate (MS222), 1 × PSF (Abcam) in 1 × MMR, pH 7.6–7.8) and thereafter immobilized with bent 0.2-mm minutien pins (Austerlitz). The forebrain was dissected using 0.1-mm and 0.15-mm minutien pins and transferred to a 1.5-ml microcentrifuge tube containing 100 µl of *Xenopus* culture media (1:100 (v/v) dilution of PSF in 6 parts L-15 with 4 parts double-distilled H_2_O) on ice. Five brains were collected per tube and upon settling at the bottom of the tube, the media was removed. Samples were resuspended in 100 µl of 1 × Ca^2+^- and Mg^2+^-free MMR, with 1% DNase (Roche). The tissues were mechanically dissociated by pipetting them 100 times and incubated at room temperature for 5 min before adding 400 µl of *Xenopus* culture media and centrifuging the solution at 400*g* for 3 min. The supernatant was removed and the pellet was resuspended in 1 ml of *Xenopus* culture media. To functionalize 35-mm glass-bottomed dishes (Ibidi), 10 µg ml^−1^ poly-D-lysine (PDL, *M*_w_ 70–150 kDa) in 1 × PBS was added for 30 min, followed by 5 µg ml^−1^ laminin in 1 × PBS for 30 min. Functionalized dishes were washed with *Xenopus* tissue culture media before adding 500 µl of dissociated cell solution and leaving them to adhere at room temperature for 15 min, before cellular stiffness was measured with an atomic force microscope.

#### Single cell stiffness measurements

Force–distance spectroscopy was performed on a JPK-Bruker CellHesion 200 mounted on a Leica DMi8 inverted microscope, with Nanoworld Arrow TL1 cantilevers. The spring constants of the cantilevers were determined using the thermal noise method in the JPK software. Cantilevers with spring constants of 0.044–0.1 N m^−1^ were selected for this study and a 5.12-µm-radius bead was glued to each. The setpoint used to measure cells was 500 pN. Only cells that did not contact other cells and were rounded in appearance (suggesting they had not spread on the glass surface) were measured. Data were processed as described in ‘Quantification of the reduced apparent elastic modulus *K*’. An outlier analysis (ROUT; *Q* = 1%) was performed using GraphPad Prism software, and outliers were excluded from further analysis.

#### Fabrication of 3D collagen hydrogels

Preparation of collagen hydrogels was adapted from ref. ^43^. Briefly, 25 mM HEPES buffer was dissolved in Leibovitz L-15 medium without L-glutamine and 100 × PSF solution (Abcam) added to achieve a final concentration of 1:100 (v/v). The solution was sterilized with a Nalgene Rapid-Flow PES 0.45-μm filter (Thermo Fisher Scientific), and the mixture was stored at 4 °C (for no longer than 2 weeks). Hydrogel premixes were prepared on ice from 6 parts L-15/HEPES/PSF and 1 part (soft gel) or 3 parts (stiff gel) 10 mg ml^−1^ bovine collagen stock solution (CellSystems). The remaining volume was filled with autoclaved double-distilled water. Polymerization of collagen hydrogels was initiated by neutralizing the pH to 7.3 with NaOH and leaving the gels at room temperature. After 2 h, solid gels were overlaid with *Xenopus* culture medium (1:100 v/v dilution of PSF in 6 parts L-15 with 4 parts double-distilled H_2_O) and incubated at 20 °C.

#### Ex vivo tissue culture in 3D collagen hydrogels

While premixes of soft and stiff collagen hydrogels were prepared on ice as described above, the hypothalamus and telencephalon brain regions were dissected from stage 37/38 embryos and placed in separate dishes containing *Xenopus* culture medium (1:100 v/v dilution of PSF in 6 parts L-15 with 4 parts double-distilled H2O). Around 1 ml of liquid hydrogel solution was then pipetted into a 35-mm glass-bottom dish (Ibidi) and left for prepolymerization at room temperature for ∼20 s. Around ten brain regions were transferred evenly throughout the solidifying gel. The dish was then kept at 20 °C for 2 h, after which an additional 2 ml of *Xenopus* culture medium was added and brain regions in hydrogels were cultured until 24 h.

#### 3D traction force microscopy

Brain tissue explants from stage 37/38 *Xenopus* embryos were dissected from the hypothalamus region, embedded in soft and stiff collagen hydrogels, and cultured for 24 h at 20 °C (as described in ‘Ex vivo tissue culture in 3D collagen hydrogels’). After 24 h, fresh *Xenopus* culture medium was added. The samples were imaged using an upright confocal laser scanning microscope (TCS SP5, Leica Microsystems) equipped with a 20× water immersion objective (HCX APO L, Leica Microsystems; numerical aperture, 1.00). Three image stacks (493 µm × 493 µm × 75 µm) with a voxel size of 481 nm × 481 nm × 2.5 µm, were acquired at 5-min intervals in reflection mode (pinhole diameter, 2 Airy units), visualizing the collagen fibre network, using the 488-nm laser line. Following the acquisition of these images, 1 mM cytochalasin D in DMSO was added to the medium at 1:125 (v/v), and another time series was acquired with four image stacks (5 min apart).

#### 3D traction force microscopy analysis

Analysis of collagen fibre deformations in 3D confocal *z* stacks was performed using the open-source Python package Saenopy^43^. Stacks acquired prior to cytochalasin D-induced tissue relaxation were drift-corrected relative to the last stack recorded after cytochalasin D-treatment. Particle image velocimetry was used for detection of deformation fields using a window size of 30 µm and an element size of 15 µm (ref. ^43^). Displacement vectors with magnitudes 40% larger than their nearest neighbours were excluded and the resulting fields interpolated to an element size of 10 µm. To solve the inverse problem, material parameters for soft (*k*_0_ = 2165, *d*_0_ = 0.0022, *λ*_s_ = 0.0093 and *d*_s_ = 0.018) and stiff (*k*_0_ = 10076, *d*_0_ = 0.0022, *λ*_s_ = 0.0056 and *d*_s_ = 0.027) collagen matrices were used as determined before^43^. For the regularization, an α parameter of 10^8.5^ with a step size of 0.33 and a fixed number of 150 iterations was used. Displacement and force magnitudes were determined using the Euclidean lengths of individual vectors. Outliers in the reconstructed displacement field were excluded based on a *z*-score threshold exceeding 4, where the *z* score for each data point was calculated as

$$z=\frac{x-{\rm{\mu }}}{\sigma }.$$

Here, *x* represents a single vector magnitude, *µ* is the mean and *σ* is the standard deviation. The strain energy^43,79^ was calculated for the whole stack volume, which corresponds to the total work performed by the tissue to deform its surroundings from the reference state. To reduce variability, scalar quantities were calculated for all vector fields derived from the three image frames of a single explant relative to the last reference frame and the median value determined.

#### Statistics and Reproducibility

No statistical method was used to predetermine sample size. No data were excluded from analyses except in Fig. 4e, where an outlier analysis (ROUT; *Q* = 1%) was performed using GraphPad Prism software, and outliers were excluded from further analysis. Names of the statistical tests used, numbers of technical and biological replicates, and *P*-values are provided in the figure captions and in Supplementary Table 1. All statistical analyses testing a null-hypothesis were two-tailed. All data were obtained from at least three independent biological replicates. Raw images analysed in Fig. 5k,l and Extended Data Fig. 6c,d were renamed to random numbers before further processing, as described in Methods (‘HCR-FISH quantification’).

### Reporting summary

Further information on research design is available in the Nature Portfolio Reporting Summary linked to this article.

## Online content

Any methods, additional references, Nature Portfolio reporting summaries, source data, extended data, supplementary information, acknowledgements, peer review information; details of author contributions and competing interests; and statements of data and code availability are available at 10.1038/s41563-025-02463-9.

## Supplementary information

Supplementary Information Supplementary video legends and Supplementary Table 1.

Reporting Summary

Supplementary Video 1 **Long-term physical interactions between a brain tissue explant and a soft hydrogel matrix**. Brain tissue from the hypothalamic region of a developing Xenopus brain was cultured in a soft collagen hydrogel for 24 hours. Maximum intensity-projected time-lapse acquisition (5 minutes between frames) of confocal stacks in reflection mode revealed dynamic matrix deformations at the tissue – fibre network interface. Scale bar: 40 µm.

Supplementary Video 2 **Traction force microscopy of brain explants in hydrogel matrices**. Brain tissue explants were cultured in soft and stiff collagen hydrogels for 24 hours. Three confocal reflection stacks were acquired every 5 minutes prior to inducing tissue relaxation by the application of Cytochalasin D, and another four stacks acquired immediately after. Here, the maximum intensity projection of a hypothalamic tissue explant in a soft collagen hydrogel is shown. White arrows indicate neurites which extended from the explant; the dashed white line marks the tissue boundary. The 3D displacement field was calculated between the first and last image stack and sum-projected along the z-axis to obtain a 2D representation with colour indicating the vector magnitudes. Scale bar: 150 µm.

## Source data

Source Data Figs. 1–6 and Extended Data Figs. 1–9 Source data.

Source Data Figs. 2 and 4 and Extended Data Fig. 5 Labelled gels or blots.

## Data Availability

The data supporting the findings of this study are available within the Article and its Supplementary Information. Source data are provided with this paper. Any other information is available from the corresponding authors upon request.

## Code availability

All code used in this paper are publicly available from Zenodo via 10.5281/zenodo.17701972 (ref. ^77^) and 10.5281/zenodo.17702192 (ref. ^78^).
