# Supplementary material for: Long-range chemical signalling in vivo is regulated by mechanical signals
Source: Nat Mater. 2026 Jan 19;25(4):687–97. doi: 10.1038/s41563-025-02463-9 (PMC13046474; doi:10.1038/s41563-025-02463-9)
Supplement: Supplementary file 1 — Supplementary video legends and Supplementary Table 1. [file 41563_2025_2463_MOESM1_ESM.pdf]

nature materials

Supplementary information https://doi.org/10.1038/s41563-025-02463-9

Long-range chemical signalling in vivo is regulated by mechanical signals

In the format provided by the authors and unedited

Supplementary Material

Table of contents

Supplementary Table 2

Supplementary table 1: Statistics in this study...3

Supplementary Table

Table 1

Figure	Panel	Statistical test	P value	One/two tailed	Degrees of freedom	Test statistic (F,t,r)	Effect size	R squared value
1	g	Kruskal-Wallis test<br>and<br>Dunns post-hoc test	p < 0.0001<br>(Adjusted p values after Dunn's post-hoc test are provided in the figure)	N/A	df = 3	H = 23.8	η^2 = 0.234	refer to η^2 value
	h	Chi-Squared test followed by Fisher's Exact post-hoc tests	p = 1.014E-8<br>(Adjusted p values after Fisher's Exact post-hoc test are provided in the figure)	Chi-Squared test: one tailed<br>Fisher's Exact post-hoc tests: two tailed	df = 6	Chi squared = 48.332	Cramers V = 0.465	-
2	d	unpaired t-test with Welch's correction	p = 0.008	Two tailed	Welch-corrected df = 21.7	Welch-corrected t = 2.93	mean difference ± SEM (95% confidence interval): -0.0119 ± 0.00406(-0.0203 to -0.00345)	R^2 = 0.283
	h	unpaired t-test with Welch's correction	p = 0.025	Two tailed	Welch-corrected df = 27.68	Welch corrected t = 2.362	mean difference ± SEM (95% confidence interval): -2.299 ± 0.9733(-4.294 to -0.3044)	R^2 = 0.1678
	l	ratio paired t-test	p = 0.0025	Two tailed	df = 3	t = 9	Geometric mean of ratios = 0.3920<br>SEM of log(ratios) = 0.04279<br>(95% confidence interval) = 0.2865 to 0.5364	R^2 = 0.9679
3	d	Kruskal-Wallis test<br>and<br>Dunns post-hoc test	p < 0.0001<br>(Adjusted p values after Dunn's post-hoc test are provided in the figure)	N/A	df = 3	H = 374.0933	η^2 = 0.1006	refer to η^2 value
	g	Wilcoxon rank sum test	p = 1.5E-4	Two tailed	N/A	U = 51589	r = 0.1554	~ r^2 = 0.0241
	h	Wilcoxon rank sum test	p = 0.656	Two tailed	N/A	U = 24322	r = 0.0211	~ r^2 = 4E-4
4	b (Rostral)	one-way ANOVA	p = 0.367	N/A	3 (between columns),<br>44 (within columns)	F = 1.08		R^2 = 0.0734
	b (Caudal)	one-way ANOVA	p = 0.454	N/A	3 (between columns),<br>44 (within columns)	F = 0.891		R^2 = 0.0612
	d	ratio paired t-test	p = 0.165	Two tailed	df = 3	t = 1.823	Geometric mean of ratios = 0.7702<br>SEM of log(ratios) = 0.06221<br>(95% confidence interval) = 0.4882 to 1.215	R^2 = 0.5254
	e	Mann-Whitney test	p = 0.14	Two tailed		Mann-Whitney U = 3558		
	f	ratio paired t-test	p = 0.0073	Two tailed	df = 2	t = 11.64	Geometric mean of ratios = 0.1527<br>SEM of log(ratios) = 0.07014<br>(95% confidence interval) = 0.07623 to 0.3060	R^2 = 0.9854
	g	ratio paired t-test	p = 0.0193	Two tailed	df = 2	t = 7.099	Geometric mean of ratios = 0.4342<br>SEM of log(ratios) = 0.05103<br>(95% confidence interval) = 0.2619 to 0.7200	R^2 = 0.9618
	i	nested t-test	p = 0.0171	Two tailed	df = 27	t = 2.54, F = 6.46	mean difference ± SEM (95% confidence interval): -74.3 ± 29.2 Pa (-134 to -14.3 Pa)	
	l	ratio paired t-test	p = 0.0102	Two tailed	df = 2	t = 9.846	Geometric mean of ratios = 0.4086<br>SEM of log(ratios) = 0.03948<br>(95% confidence interval) = 0.2763 to 0.6042	R^2 = 0.9798
5	e	Mann-Whitney U test	p = 0.024	Two tailed	N/A	U = 352		N/A
	f	Mann-Whitney U test	p = 2.01E-7	Two tailed	N/A	U = 23		N/A
	g	Mann-Whitney U test	p = 1.34E-6	Two tailed	N/A	U = 39		N/A
	k	ratio paired t-test	p = 0.0245	Two tailed	df = 4	t = 3.517	Geometric mean of ratios = 1.458<br>SEM of log(ratios) = 0.04660<br>(95% confidence interval) = 1.083 to 1.965	R^2 = 0.7556
	l	ratio paired t-test	p = 0.050	Two tailed	df = 4	t = 2.776	Geometric mean of ratios = 1.307<br>SEM of log(ratios) = 0.04185<br>(95% confidence interval) = 0.9999 to 1.708	R^2 = 0.6582
6	d	unpaired t-test with Welch's correction	p = 0.0111	Two tailed	Welch-corrected df = 10.61	Welch-corrected t = 3.070	mean difference ± SEM (95% confidence interval): 1.440 ± 0.4688 (0.4029 to 2.476)	R^2 = 0.4706
	h	Kruskal-Wallis test<br>and<br>Dunns post-hoc test	p < 0.0001<br>(Adjusted p values after Dunn's post-hoc test are provided in the figure)			Kruskal-Wallis statisitc<br>=<br>17.74		
**Figure**	**Panel**	**Statistical test**	**P value**	**One/two tailed**	**Degrees of freedom**	**Test statistic (F,t,r)**	**Effect size**	**R squared value**
Supplementary Figure 1	e	ratio paired t-test	p = 0.027	Two tailed	df = 3	t = 4.026	Geometric mean of ratios =0.5493<br>SEM of log(ratios) = 0.06463<br>(95% confidence interval) = 0.3421 to 0.8821	R^2 = 0.8438
Supplementary Figure 3	a	ratio paired t-test	p = 0.0128	Two tailed	df = 3	t = 5.343	Geometric mean of ratios = 0.3694<br>SEM of log(ratios) = 0.08095<br>(95% confidence interval) = 0.2041 to 0.6685	R^2 = 0.9049
	b	ratio paired t-test	p = 0.4095	Two tailed	df = 3	t = 0.9563	Geometric mean of ratios = 0.8446<br>SEM of log(ratios) = 0.07671<br>(95% confidence interval) = 0.4814 to 1.482	R^2 = 0.2336
Supplementary Figure 4	c	nested t-test	p = 0.0001	Two tailed	df = 24	t = 4.539 , F = 20.60	mean difference ± SEM (95% confidence interval): -22.78 ± 5.019 Pa (-33.14 Pa to -12.42 Pa)	
	a	ratio paired t-test	p = 0.0451	Two tailed	df = 3	t = 3.319	Geometric mean of ratios = 0.5455<br>SEM of log(ratios) = 0.07928<br>(95% confidence interval) = 0.3051 to 0.9753	R^2 = 0.7860
	b	ratio paired t-test	p = 0.006	Two tailed	df = 3	t = 6.976	Geometric mean of ratios = 0.3976<br>SEM of log(ratios) = 0.05743<br>(95% confidence interval) = 0.2610 to 0.6056	R^2 = 0.9419
	c	ratio paired t-test	p = 0.1988	Two tailed	df = 2	t = 1.894	Geometric mean of ratios = 0.6312<br>SEM of log(ratios) = 0.1055<br>(95% confidence interval) = 0.2219 to 1.795	R^2 = 0.6420
Supplementary Figure 5	e	ratio paired t-test	p = 0.0116	Two tailed	df = 2	t = 9.214	Geometric mean of ratios = 0.02215<br>SEM of log(ratios) = 0.1796<br>(95% confidence interval) = 0.003740 to 0.1312	R^2 = 0.9770

	f	ratio paired t-test	p = 0.0008	Two tailed	df = 2	t = 36.08	Geometric mean of ratios = 0.2733<br>SEM of log(ratios) = 0.01561<br>(95% confidence interval) = 0.2342 to 0.3190	R^2 = 0.9985
	g	ratio paired t-test	p = 0.157	Two tailed	df = 2	t = 2.216	Geometric mean of ratios = 0.5468<br>SEM of log(ratios) = 0.1183<br>(95% confidence interval) = 0.1694 to 1.766	R^2 = 0.7105
	h	ratio paired t-test	p = 0.227	Two tailed	df = 2	t = 1.724	Geometric mean of ratios = 0.8327<br>SEM of log(ratios) = 0.04611<br>(95% confidence interval) = 0.5274 to 1.315	R^2 = 0.5978
Supplementary Figure 6	c	ratio paired t-test	p = 0.8237	Two tailed	df = 4	t = 0.2378	Geometric mean of ratios = 1.040<br>SEM of log(ratios) = 0.07181<br>(95% confidence interval) = 0.6572 to 1.646	R^2 = 0.01394
	d	ratio paired t-test	p = 0.5416	Two tailed	df = 4	t = 0.6664	Geometric mean of ratios = 1.093<br>SEM of log(ratios) = 0.05819<br>(95% confidence interval) = 0.7538 to 1.586	R^2 = 0.09993
Supplementary Figure 7	c	nested t-test	p = 0.01	Two tailed	df = 7	t = 3.4	mean difference ± SEM (95% confidence interval): 126 ± 37 Pa (39 Pa to 213 Pa)	
	f	unpaired t-test with Welch's correction	p = 0.05	Two tailed	Welch-corrected df = 5.998	Welch-corrected t = 2.430	mean difference ± SEM (95% confidence interval): 0.02109 ± 0.008678(-0.0001494 to 0.04232)	R^2 = 0.4961
Supplementary Figure 8	e	repeated measures one-way ANOVA<br>with Geisser-Greenhouse correction,<br>followed by a Tukey's multiple comparison test	p values are provided in the figure panel		3 (between columns),<br>9 (between rows)	F = 149		R^2 = 0.943
	h	repeated measures one-way ANOVA<br>with Geisser-Greenhouse correction,<br>followed by a Tukey's multiple comparison test	p values are provided in the figure panel		2 (between columns),<br>9 (between rows)	F = 72.6		R^2 = 0.890
	i	one sample t-test	p = 0.499	Two-tailed	df = 4	t = 0.742		R^2 = 0.121
Supplementary Figure 9	d	unpaired t-test with Welch's correction	p = 0.1685	Two-tailed	Welch-corrected df = 16.69	Welch-corrected t = 1.440	mean difference ± SEM (95% confidence interval): -0.5200 ± 0.3612 (-1.283 to 0.2431)	R^2 = 0.1105

Supplementary table 1: Statistics in this study

The table shows the statistical tests used in this study, the p-values obtained, degrees of freedom, test statistic value, effect sizes, and R squared values of each experiment conducted.
