# Supplementary material for: Long-range chemical signalling in vivo is regulated by mechanical signals
Source: Nat Mater. 2026 Jan 19;25(4):687–97. doi: 10.1038/s41563-025-02463-9 (PMC13046474; doi:10.1038/s41563-025-02463-9)
Supplement: Supplementary file 2 — Reporting Summary [file 41563_2025_2463_MOESM2_ESM.pdf]

nature portfolio

Corresponding author(s): E.K.P, S.M. and K.F

Last updated by author(s): Oct 21, 2025

# Reporting Summary

Nature Portfolio wishes to improve the reproducibility of the work that we publish. This form provides structure for consistency and transparency in reporting. For further information on Nature Portfolio policies, see our Editorial Policies and the Editorial Policy Checklist.

## Statistics

For all statistical analyses, confirm that the following items are present in the figure legend, table legend, main text, or Methods section.

| n/a | Confirmed | |
|---|---|---|
| ☐ | ☒ | The exact sample size (*n*) for each experimental group/condition, given as a discrete number and unit of measurement |
| ☐ | ☒ | A statement on whether measurements were taken from distinct samples or whether the same sample was measured repeatedly |
| ☐ | ☒ | The statistical test(s) used AND whether they are one- or two-sided<br>*Only common tests should be described solely by name; describe more complex techniques in the Methods section.* |
| ☐ | ☒ | A description of all covariates tested |
| ☐ | ☒ | A description of any assumptions or corrections, such as tests of normality and adjustment for multiple comparisons |
| ☐ | ☒ | A full description of the statistical parameters including central tendency (e.g. means) or other basic estimates (e.g. regression coefficient) AND variation (e.g. standard deviation) or associated estimates of uncertainty (e.g. confidence intervals) |
| ☐ | ☒ | For null hypothesis testing, the test statistic (e.g. *F*, *t*, *r*) with confidence intervals, effect sizes, degrees of freedom and *P* value noted<br>*Give P values as exact values whenever suitable.* |
| ☒ | ☐ | For Bayesian analysis, information on the choice of priors and Markov chain Monte Carlo settings |
| ☒ | ☐ | For hierarchical and complex designs, identification of the appropriate level for tests and full reporting of outcomes |
| ☒ | ☐ | Estimates of effect sizes (e.g. Cohen's *d*, Pearson's *r*), indicating how they were calculated |

*Our web collection on statistics for biologists contains articles on many of the points above.*

## Software and code

Policy information about availability of computer code

| | |
|---|---|
| Data collection | *Provide a description of all commercial, open source and custom code used to collect the data in this study, specifying the version used OR state that no software was used.* |
| Data analysis | available at https://github.com/FranzeLab |

For manuscripts utilizing custom algorithms or software that are central to the research but not yet described in published literature, software must be made available to editors and reviewers. We strongly encourage code deposition in a community repository (e.g. GitHub). See the Nature Portfolio guidelines for submitting code & software for further information.

## Data

Policy information about availability of data

All manuscripts must include a data availability statement. This statement should provide the following information, where applicable:

- Accession codes, unique identifiers, or web links for publicly available datasets
- A description of any restrictions on data availability
- For clinical datasets or third party data, please ensure that the statement adheres to our policy

Data availability statement included.

## Research involving human participants, their data, or biological material

Policy information about studies with human participants or human data. See also policy information about sex, gender (identity/presentation), and sexual orientation and race, ethnicity and racism.

| | |
|---|---|
| Reporting on sex and gender | *Use the terms sex (biological attribute) and gender (shaped by social and cultural circumstances) carefully in order to avoid confusing both terms. Indicate if findings apply to only one sex or gender; describe whether sex and gender were considered in study design; whether sex and/or gender was determined based on self-reporting or assigned and methods used. Provide in the source data disaggregated sex and gender data, where this information has been collected, and if consent has been obtained for sharing of individual-level data; provide overall numbers in this Reporting Summary. Please state if this information has not been collected. Report sex- and gender-based analyses where performed, justify reasons for lack of sex- and gender-based analysis.* |
| Reporting on race, ethnicity, or other socially relevant groupings | *Please specify the socially constructed or socially relevant categorization variable(s) used in your manuscript and explain why they were used. Please note that such variables should not be used as proxies for other socially constructed/relevant variables (for example, race or ethnicity should not be used as a proxy for socioeconomic status). Provide clear definitions of the relevant terms used, how they were provided (by the participants/respondents, the researchers, or third parties), and the method(s) used to classify people into the different categories (e.g. self-report, census or administrative data, social media data, etc.) Please provide details about how you controlled for confounding variables in your analyses.* |
| Population characteristics | *Describe the covariate-relevant population characteristics of the human research participants (e.g. age, genotypic information, past and current diagnosis and treatment categories). If you filled out the behavioural & social sciences study design questions and have nothing to add here, write "See above."* |
| Recruitment | *Describe how participants were recruited. Outline any potential self-selection bias or other biases that may be present and how these are likely to impact results.* |
| Ethics oversight | *Identify the organization(s) that approved the study protocol.* |

Note that full information on the approval of the study protocol must also be provided in the manuscript.

# Field-specific reporting

Please select the one below that is the best fit for your research. If you are not sure, read the appropriate sections before making your selection.

☒ Life sciences ☐ Behavioural & social sciences ☐ Ecological, evolutionary & environmental sciences

For a reference copy of the document with all sections, see nature.com/documents/nr-reporting-summary-flat.pdf

# Life sciences study design

All studies must disclose on these points even when the disclosure is negative.

| | |
|---|---|
| Sample size | Disclosed within the text and in legends. |
| Data exclusions | No data were excluded from analyses except in Figure 4e, where an outlier analysis (ROUT; Q = 1%) was performed using GraphPad PRISM software, and outliers were excluded from further analysis. |
| Replication | Biological and technical replicates included, numbers are described clearly in figures/methods. |
| Randomization | Where possible, images were randomized prior to analysis. Disclosed in the text. |
| Blinding | Not possible due to the obvious phenotype of mutants, however all conditions in imaging/analysis were maintained between different conditions, and images were analysed blinded where possible. Disclosed in the text. |

# Reporting for specific materials, systems and methods

We require information from authors about some types of materials, experimental systems and methods used in many studies. Here, indicate whether each material, system or method listed is relevant to your study. If you are not sure if a list item applies to your research, read the appropriate section before selecting a response.

## Materials & experimental systems

| n/a | Involved in the study |
| --- | --- |
| ☐ | ☒ Antibodies |
| ☒ | ☐ Eukaryotic cell lines |
| ☒ | ☐ Palaeontology and archaeology |
| ☐ | ☒ Animals and other organisms |
| ☒ | ☐ Clinical data |
| ☒ | ☐ Dual use research of concern |
| ☒ | ☐ Plants |

## Methods

| n/a | Involved in the study |
| --- | --- |
| ☒ | ☐ ChIP-seq |
| ☒ | ☐ Flow cytometry |
| ☒ | ☐ MRI-based neuroimaging |

# Antibodies

Antibodies used

Primary antibodies: anti-FAM38A (NBP1-78446), anti-sema3A (ab199475), anti-beta-actin (ab6276), anti-NCAM1 (DSHB Cat#4d, RRID:AB_528389), anti-N-cadherin (AF6426-SP), anti-α tubulin (acetyl K40; ab179484), anti-α tubulin (ab7291), anti-Piezo1 antibody (28511-1-AP)

Validation

anti-FAM38A (NBP1-78446): verified by decrease in western blot band intensity in Piezo1 downregulated tissues (Fig. 2k, SupFig. 3a) using morpholinos. Morpholinos were previously verified for downregulation effect with a different antibody (PMID: 27643431).

anti-sema3A (ab199475): verified by decrease in western blot band intensity in Sema3A downregulated tissues using morpholinos. Morpholinos were previously independently verified for downregulation in PMID: 23292679.

anti-beta-actin (ab6276): verified with manufacturer 100% homology to Xenopus sequence.

anti-NCAM1 (DSHB Cat#4d, RRID:AB_528389), confirmed species reactivity listed on Developmental Studies Hybridoma Bank (DSHB, https://dshb.biology.uiowa.edu/4d), first used in Xenopus (PMID: 8972224).

anti-N-cadherin (AF6426-SP): verified by decrease in western blot band intensity in NCAD downregulated tissues (SupFig. 5f) using previously used morpholinos (PMID: 19279134).

anti-α tubulin (ab7291): verified for use in PMID: PMC7904641.

anti-α tubulin (acetyl K40; ab179484): verified use in Xenopus in PMID: 39131273.

anti-Piezo1 antibody (28511-1-AP): verified by decrease in immunohistochemistry signal (see SupFig. 1) using morpholinos. Morpholinos were previously verified for downregulation effect with a different antibody (PMID: 27643431).

# Animals and other research organisms

Policy information about studies involving animals; ARRIVE guidelines recommended for reporting animal research, and Sex and Gender in Research

Laboratory animals

Wild type Xenopus laevis embryos were used in all experiments. Knockdown embryos were generated through microinjections at early stages. Staging is based on the Nieuwkoop and Faber tables.

Wild animals

N/A

Reporting on sex

Embryos of both sexes were used in all experiments.

Field-collected samples

N/A

Ethics oversight

All animal experiments were approved by the Ethical Review Committee of the University of Cambridge, in compliance with guidelines set by the UK Home Office.

Note that full information on the approval of the study protocol must also be provided in the manuscript.

## Plants

| | |
|---|---|
| Seed stocks | *Report on the source of all seed stocks or other plant material used. If applicable, state the seed stock centre and catalogue number. If plant specimens were collected from the field, describe the collection location, date and sampling procedures.* |
| Novel plant genotypes | *Describe the methods by which all novel plant genotypes were produced. This includes those generated by transgenic approaches, gene editing, chemical/radiation-based mutagenesis and hybridization. For transgenic lines, describe the transformation method, the number of independent lines analyzed and the generation upon which experiments were performed. For gene-edited lines, describe the editor used, the endogenous sequence targeted for editing, the targeting guide RNA sequence (if applicable) and how the editor was applied.* |
| Authentication | *Describe any authentication procedures for each seed stock used or novel genotype generated. Describe any experiments used to assess the effect of a mutation and, where applicable, how potential secondary effects (e.g. second site T-DNA insertions, mosiacism, off-target gene editing) were examined.* |
