# Supplementary figures and images for: Long-range chemical signalling in vivo is regulated by mechanical signals
Source: Nat Mater. 2026 Jan 19;25(4):687–97. doi: 10.1038/s41563-025-02463-9 (PMC13046474; doi:10.1038/s41563-025-02463-9)

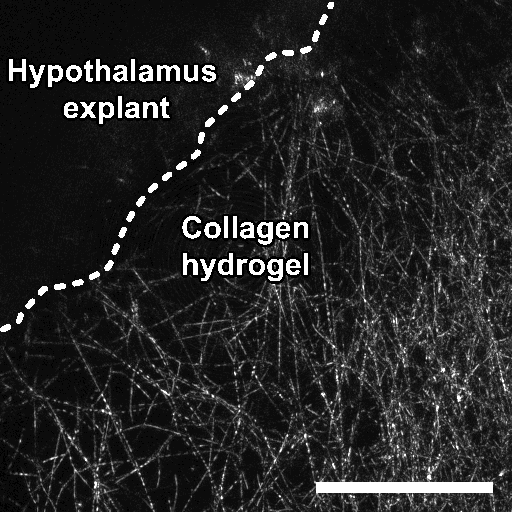

Supplement: Supplementary file 3 — Long-term physical interactions between a brain tissue explant and a soft hydrogel matrix. Brain tissue from the hypothalamic region of a developing Xenopus brain was cultured in a soft collagen hydrogel for 24 hours. Maximum intensity-projected time-lapse acquisition (5 minutes between frames) of confocal stacks in reflection mode revealed dynamic matrix deformations at the tissue – fibre network interface. Scale bar: 40 µm. [file 41563_2025_2463_MOESM3_ESM.gif]

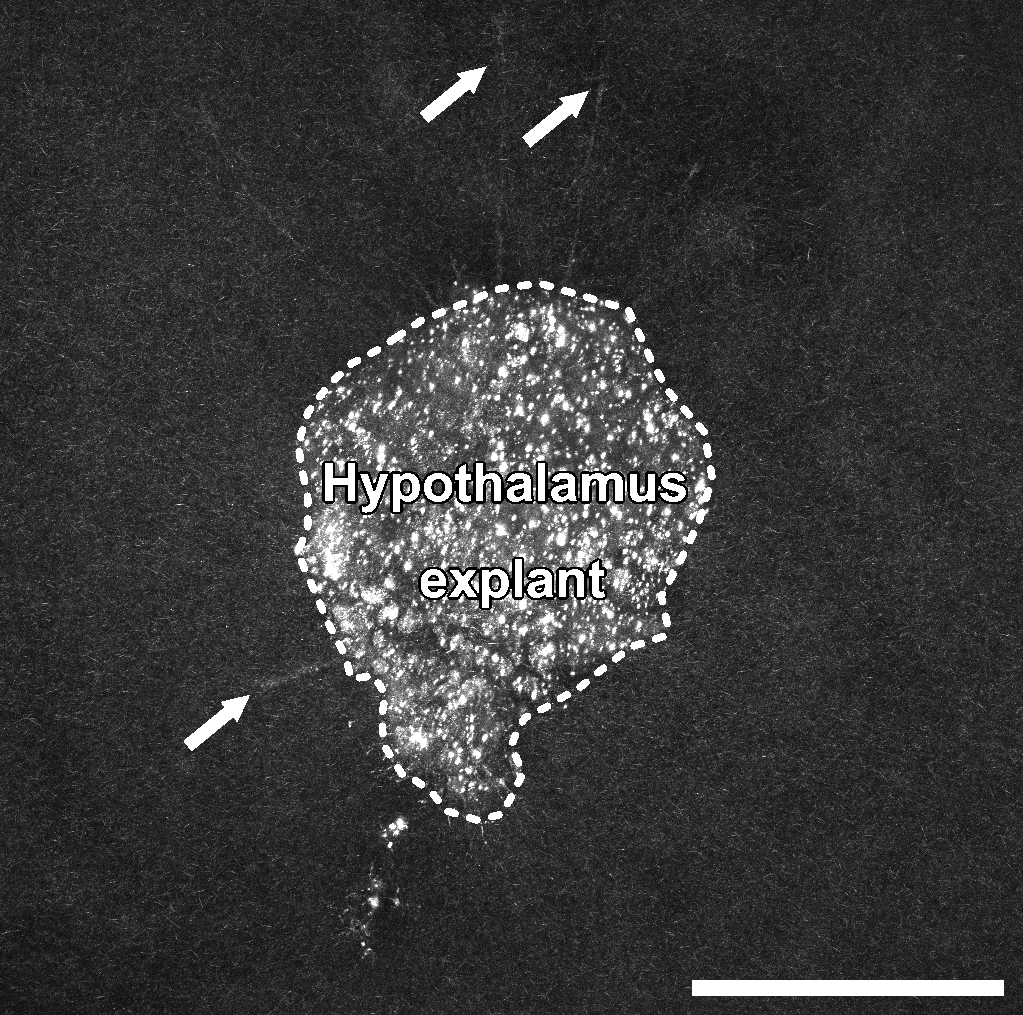

Supplement: Supplementary file 4 — Traction force microscopy of brain explants in hydrogel matrices. Brain tissue explants were cultured in soft and stiff collagen hydrogels for 24 hours. Three confocal reflection stacks were acquired every 5 minutes prior to inducing tissue relaxation by the application of Cytochalasin D, and another four stacks acquired immediately after. Here, the maximum intensity projection of a hypothalamic tissue explant in a soft collagen hydrogel is shown. White arrows indicate neurites which extended from the explant; the dashed white line marks the tissue boundary. The 3D displacement field was calculated between the first and last image stack and sum-projected along the z-axis to obtain a 2D representation with colour indicating the vector magnitudes. Scale bar: 150 µm. [file 41563_2025_2463_MOESM4_ESM.gif]
