# Supplementary material for: Long-range chemical signalling in vivo is regulated by mechanical signals
Source: Nat Mater. 2026 Jan 19;25(4):687–97. doi: 10.1038/s41563-025-02463-9 (PMC13046474; doi:10.1038/s41563-025-02463-9)
Supplement: Supplementary file 6 — Labelled gels or blots. [file 41563_2025_2463_MOESM6_ESM.pdf]

Source Data for Fig. 2k

Replicate 1

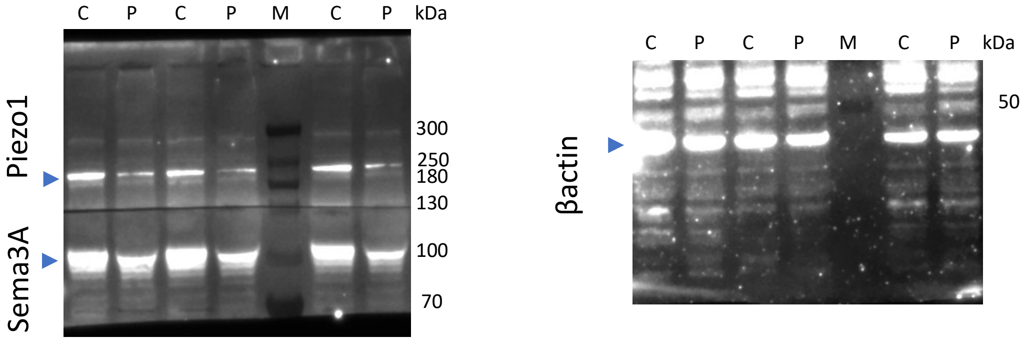

Replicate 2

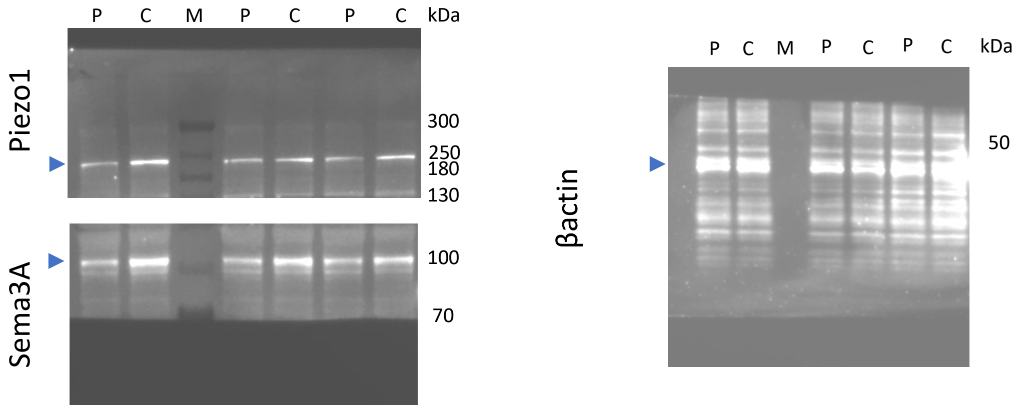

Replicate 3

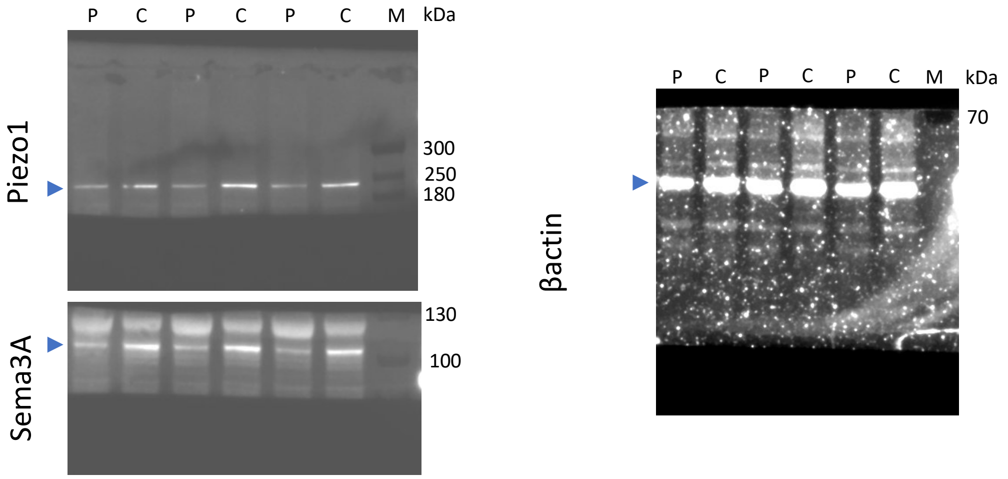

Replicate 4

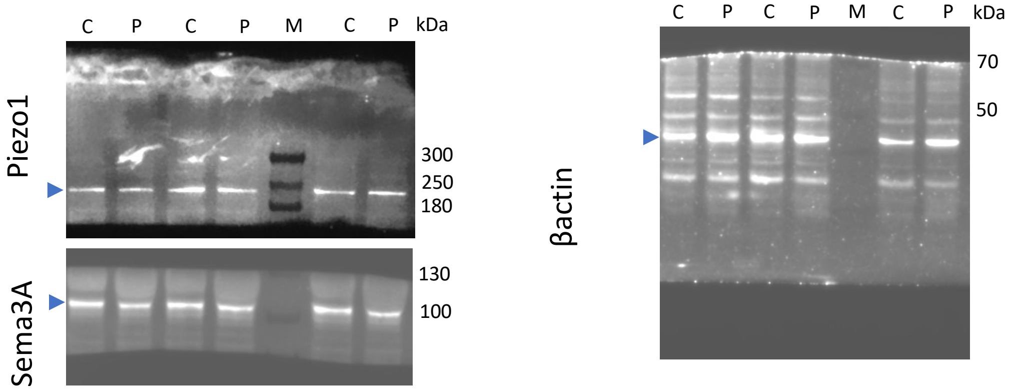

Legend

C = Control. P = Piezo1 Knockdown. M = Marker. kDa = Molecular weight of marker. ▶ Band of interest.

Source Data for Fig. 4c

Replicate 1

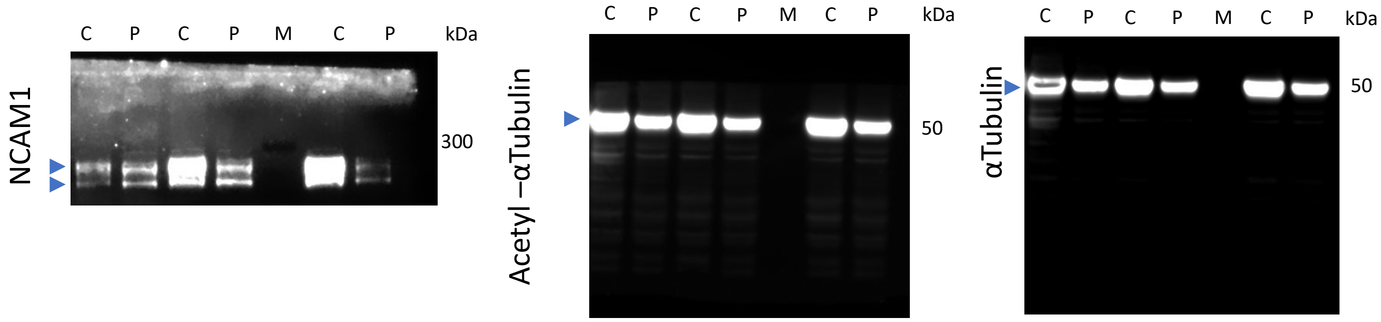

Replicate 2

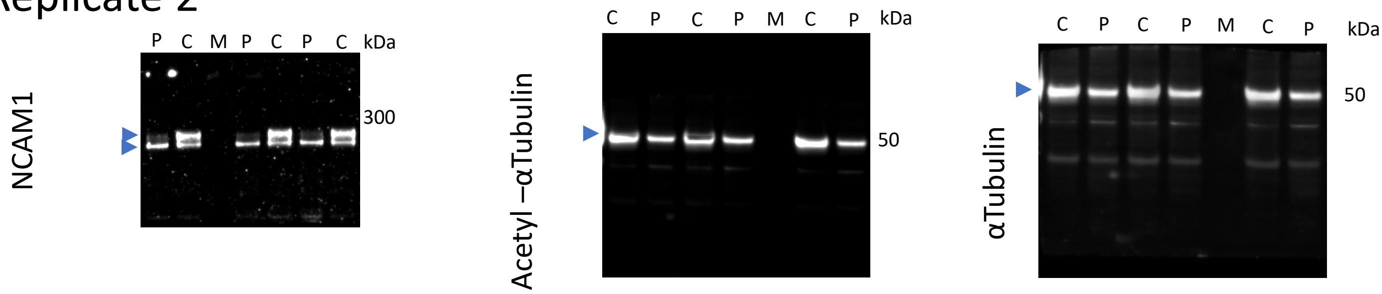

Replicate 3

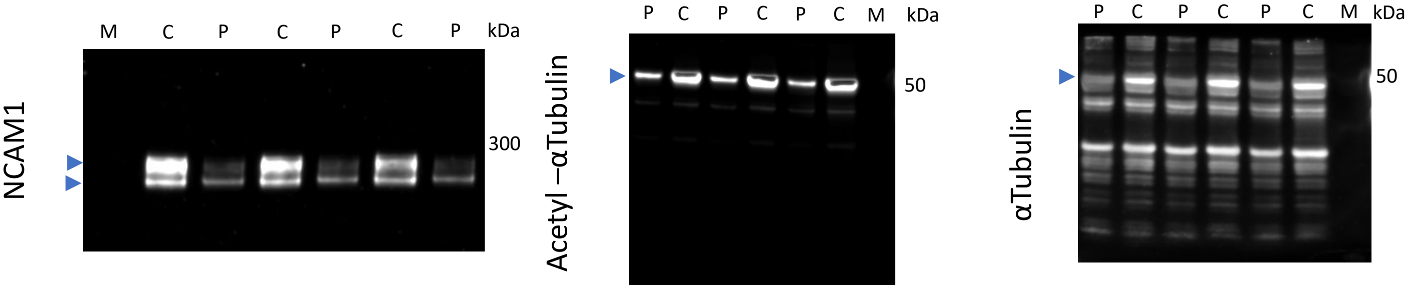

Replicate 1

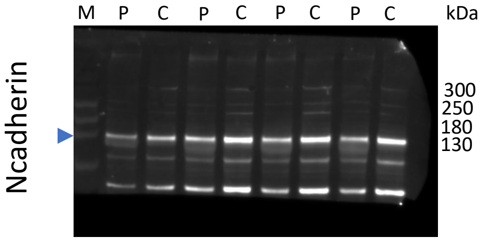

Replicate 2

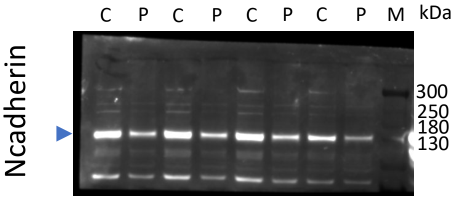

Replicate 3

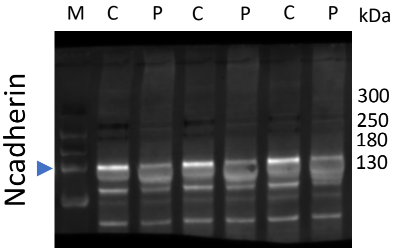

Legend

C = Control. P = Piezo1 Knockdown. M = Marker. kDa = Molecular weight of marker. ▶ Band of interest.

Source Data for Fig. 4c

Replicate 1

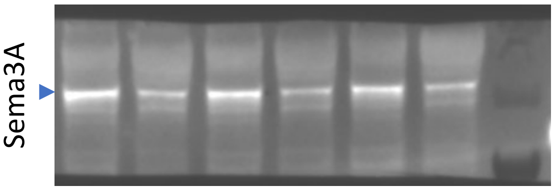

Replicate 2

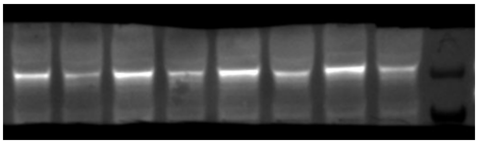

Replicate 3

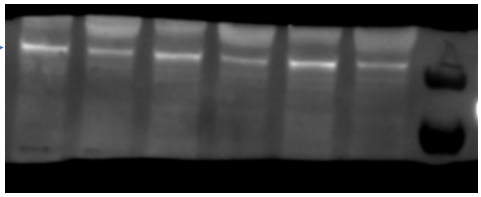

Legend

C = Control.

N = NCAM1 and N-cadherin Knockdown.

kDa = Molecular weight of marker.

M = Marker.

▶ Band of interest.

Source Data for Extended Data 5d

Replicate 1

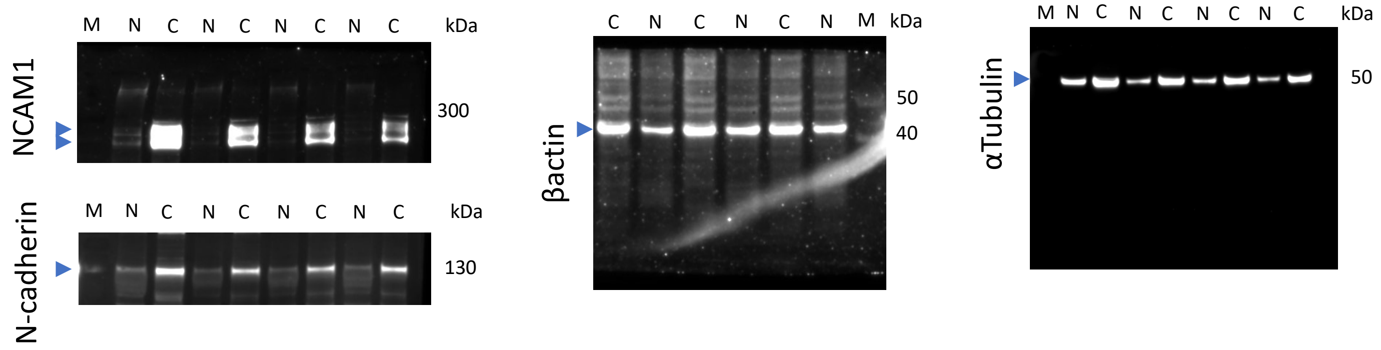

Replicate 2

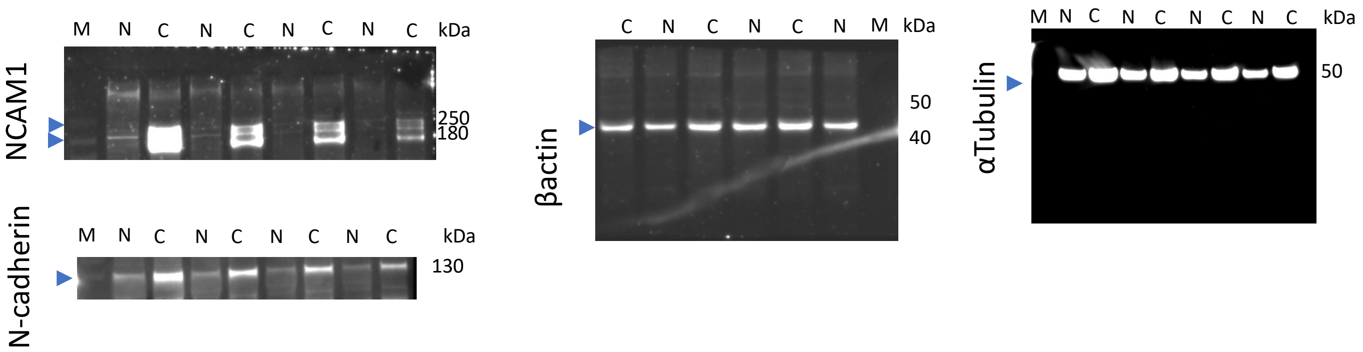

Replicate 3

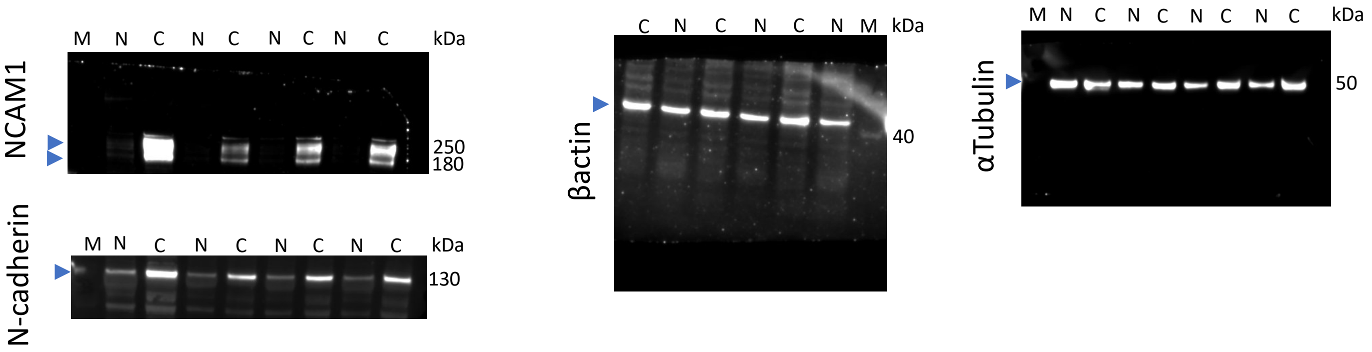

Legend

C = Control.

N = NCAM1 and N-cadherin Knockdown.

kDa = Molecular weight of marker.

M = Marker.

▶ Band of interest.
